# Macrophage and fibroblast trajectory inference and crosstalk analysis during myocardial infarction using integrated single-cell transcriptomic datasets

**DOI:** 10.1186/s12967-024-05353-x

**Published:** 2024-06-12

**Authors:** Da Ke, Mingzhen Cao, Jian Ni, Yuan Yuan, Jiangyang Deng, Si Chen, Xiujun Dai, Heng Zhou

**Affiliations:** 1 Department of Cardiology, Renmin Hospital of Wuhan University, Jiefang Road 238, Wuhan, 430060 People’s Republic of China; 2 Hubei Key Laboratory of Metabolic and Chronic Diseases, Wuhan, People’s Republic of China

**Keywords:** Myocardial infarction, scRNA-seq, Cardiac fibrosis, Fibroblasts, Macrophages

## Abstract

**Background:**

Cardiac fibrosis after myocardial infarction (MI) has been considered an important part of cardiac pathological remodeling. Immune cells, especially macrophages, are thought to be involved in the process of fibrosis and constitute a niche with fibroblasts to promote fibrosis. However, the diversity and variability of fibroblasts and macrophages make it difficult to accurately depict interconnections.

**Methods:**

We collected and reanalyzed scRNA-seq and snRNA-seq datasets from 12 different studies. Differentiation trajectories of these subpopulations after MI injury were analyzed by using scVelo, PAGA and Slingshot. We used CellphoneDB and NicheNet to infer fibroblast-macrophage interactions. Tissue immunofluorescence staining and in vitro experiments were used to validate our findings.

**Results:**

We discovered two subsets of ECM-producing fibroblasts, reparative cardiac fibroblasts (RCFs) and matrifibrocytes, which appeared at different times after MI and exhibited different transcriptional profiles. We also observed that CTHRC1^+^ fibroblasts represent an activated fibroblast in chronic disease states. We identified a macrophage subset expressing the genes signature of SAMs conserved in both human and mouse hearts. Meanwhile, the SPP1^hi^ macrophages were predominantly found in the early stages after MI, and cell communication analysis indicated that SPP1^hi^ macrophage-RCFs interactions are mainly involved in collagen deposition and scar formation.

**Conclusions:**

Overall, this study comprehensively analyzed the dynamics of fibroblast and macrophage subsets after MI and identified specific subsets of fibroblasts and macrophages involved in scar formation and collagen deposition.

**Supplementary Information:**

The online version contains supplementary material available at 10.1186/s12967-024-05353-x.

## Background

Fibrosis is a pathophysiologic process common to various cardiovascular diseases [1]. Activation of fibroblasts and deposition of extracellular matrix (ECM) proteins during the acute phase of myocardial infarction (MI) effectively prevents cardiac rupture and reduces the mortality rate [2,3]. However, persistent fibrosis alters the normal structure of the heart and leads to a decrease in cardiac function [4]. This responds to the fact that fibroblasts may have a dual role at different stages of MI. Similarly, the pathogenesis of cardiac fibrosis varies from one etiology to another, reflecting the complexity of the disease itself. Myofibroblasts are regarded as essential cells in the fibrotic process, but there is diversity in the origin of myofibroblasts, which makes it difficult to accurately profile and study this cell population [5]. Multiple immune cells and signaling pathways are involved in fibroblast activation and subsequent pathophysiologic processes. The intertwining of inflammation and fibrosis poses a significant challenge to the precise treatment of fibrosis.

Macrophages, the most abundant immune cell type in the heart [6], are extensively involved in the tissue response to injury, and different macrophage activation states play a key role in the development and resolution of fibrosis [6]. Scar-associated macrophages (SAMs), a recently defined CD9^+^TREM2^+^macrophage, have been found to have a similar phenotype in multiple tissues and organs [7–9]. In several disease models, this macrophage, which is closely associated with fibroblast activation, also expresses Spp1 and expands after organ injury [10]. Conserved monocyte-derived SAMs phenotype among different tissues and organs reflects mechanisms shared in the progression of fibrosis [11]. Several studies have also found that SAMs are spatially located close to ECM-producing fibroblasts and together constitute the fibrotic niche [12–14]. Accurate mapping of immune cell, especially macrophage, interactions during fibroblast activation is important to uncover new therapeutic targets for fibrosis treatment.

Recent applications of single-cell RNA-sequencing (scRNA-seq) technologies in cardiovascular disease have provided us with a dynamic and nuanced understanding of changes in different cellular subpopulations [12,15–20]. However, individual studies tend to focus on a particular disease state or population of cells and thus do not provide a comprehensive overview of the disease process and often lack validation in human scRNA-seq datasets. Here, we collect published mouse and human scRNA-seq datasets covering a wide range of cardiovascular diseases and different murine disease models, including multiple stages of MI, transverse aortic constriction (TAC) models, ischemic cardiomyopathy (ICM), dilated cardiomyopathy (DCM) and hypertrophic cardiomyopathy (HCM). We focus on the phenotypes of different subsets of fibroblasts and macrophages and their dynamic transition during MI. At the same time, we attempted to depict fibroblast-macrophage interactions in different disease states. Immunofluorescence staining and adenovirus transfection experiments were used to validate our findings. These data can further enhance our understanding of cardiac fibroblast properties as well as provide valuable insights for subsequent macrophage-based treatment of cardiac fibrosis.

## Methods

### scRNA-seq and snRNA-seq datasets collected in this study

scRNA-seq and snRNA-seq datasets were acquired from the Gene Expression Omnibus database (https://www.ncbi.nlm.nih.gov/geo/), ArrayExpress (https://www.ebi.ac.uk/biostudies/arrayexpress), Figshare (https://figshare.com/) and Human Cell Atlas (https://www.humancellatlas.org/) (Table 1). The mouse single-cell transcriptomic datasets used in this study included: E-MTAB-9816(MI) [19], E-MTAB-7895(MI) [16], GSE135310 (MI) [20], GSE163129 (MI) [18], GSE163465 (MI) [17], GSE185265(MI) [21], GSE155882 (TAC) [22], GSE132144(MI) [23]. The human single-cell transcriptomic datasets used in this study included: GSE145154 (normal and failed human heart) [24], a project contributed by Christoph Kuppe et al. [12], GSE185100 [25], a seurat object for snRNA-seq dataset of the cardiac tissues from 10 HCM patients and 2 healthy donors contributed by Liu et al [26]. The non-cardiomyocyte scRNA-seq datasets E-MTAB-9816 and E-MTAB-7895 contain samples from multiple time points post MI and were available as raw sequencing files, and therefore served as the main mouse datasets for our analysis (Table S1). The main human datasets were derived from partial samples from the Christoph Kuppe et al. dataset and GSE145154, collectively comprising cardiac tissue from AMI, ICM and healthy groups (Table S1). Other scRNA-seq or snRNA-seq datasets were utilized as validation datasets to verify and extend our findings, reducing the impact of limitations of a single dataset (Table S2).

**Table 1:** The scRNA-seq and snRNA-seq datasets used in this study

Datasets	Repository	Species	Platform	References	Purpose
E-MTAB-9816	ArrayExpress	Mouse	10X Genomics	Nat Commun	Main dataset
E-MTAB-7895	ArrayExpress	Mouse	10X Genomics	Cell Rep	Main dataset
GSE132144	GEO	Mouse	10X Genomics	Circulation	Validation dataset
GSE185265	GEO	Mouse	10X Genomics	Circulation	Validation dataset
GSE155882	GEO	Mouse	10X Genomics	Nature	Validation dataset
GSE135310	GEO	Mouse	10X Genomics	Circ Res	Validation dataset
GSE163129	GEO	Mouse	10X Genomics	Nat Commun	Validation dataset
GSE163465	GEO	Mouse	10X Genomics	Small Methods	Validation dataset
Kuppe et al. dataset	Human Cell Atlas	Human	10X Genomics	Nature	Main dataset
GSE145154	GEO	Human	10X Genomics	Basic Res Cardiol	Main dataset
GSE185100	GEO	Human	10X Genomics	Circulation	Validation dataset
Liu et al. dataset	Figshare	Human	10X Genomics	Cell discovery	Validation dataset

### Single-cell transcriptomic datasets processing and analysis

Raw fastq files (E-MTAB-9816, E-MTAB-7895) were obtained from the European Nucleotide Archive and were processed using CellRanger v3.0.2 (10 × Genomics). The scRNA-seq fastq data files were aligned with STAR to the mm10 genome (gencode vm23) index, annotated via GTF file and grouped by barcodes and UMIs resulting in a feature-barcode matrix.

The gene count matrix was loaded into R (4.2.3) and pre-processed using Seurat [27] (4.3.0.1). A Seurat object was generated by using CreateSeuratObject function, cells with low (≤ 500) or abnormally high (≥ 8000) gene counts and a high percentage of mitochondrial genes (≥ 20%) were removed. Subsequently, we normalize the Seurat object, find variable features, scaling and dimensionality reduction by principal-component analysis (PCA). Finally, we use harmony [28] to integrate the different Seurat object to eliminate potential batch effects. Uniform manifold approximation and projection (UMAP) dimensional reduction by ‘RunUMAP’ function was used to visualize the cell clusters across conditions. Differential expression of genes (DEGs) in each of the clusters were determined using ‘FindAllMarkers’ (logfc.threshold = 0.25, min.pct = 0.25). The protein–protein interaction (PPI) network of overlap DEGs was created based on the STRING database (http://string-db.org). The DEGs were analyzed with Enrichr [29,30] to identify enriched biological processes (Gene Ontology (GO) Biological Process 2023 in Enrichr). The signature of cell subpopulations were selected from the TOP DEGs. Here, we used the R package “AUCell” to map gene sets to human or murine single-cell datasets to discover subpopulations of cells with similar expression profiles.

### Cell–cell interaction analysis by CellPhoneDB and NicheNet

CellPhoneDB [31] is a publicly available repository of curated receptors, ligands and their interactions and can be used to search for a particular ligand/receptor. We used the CellphoneDB database to detect cell-to-cell communication in our newly generated data. First, we extracted the gene expression matrix and metadata from the integrated snRNA-seq data, and then used the statistical analysis method in CellphoneDB to analyze cell–cell interactions. For the single-cell dataset where the species is mouse, we converted the gene id to the corresponding homologue in human. Finally, we use the R package “ktplots” and “circlize” for visualization of the results.

NicheNet [32] can be used to predict cellular intercommunication. NicheNet uses human or mouse scRNA-seq datasets as inputs in combination with a database of known ligand-to-target signaling paths to infer possible interactions between different cell types. NicheNet prioritizes ligands according to their activity and looks for affected targets with high potential to be regulated by these prioritized ligands. In our study, the receiver cell population is the ‘fibroblasts’ cell population, whereas the sender cell populations are ‘macrophages’. The gene sets of interest were genes that were differentially expressed in fibroblasts in MI or ICM compared to controls.

### RNA velocity and single-cell trajectory inference

The mouse 10 × scRNA-seq data generated from E-MTAB-7895 were used for the RNA velocity analysis. First of all, BAM files were preprocessed with samtools to make them compatible with velocyto.py [33]. The different Loom files generated by velocyto.py are then loaded into scVelo [34] and integrated. We use the scv.pp.filter_and_normalize function to process the data. The moments of normalized spliced and unspliced counts were calculated using the scvelo.pp.moments function for each cell with default parameters. The RNA velocity was estimated using the scvelo.tl.velocity function with the ‘‘stochastic’’ or ‘‘dynamical’’ model, and the velocity graph was built using the scvelo.tl.velocity_graph function. The RNA velocities were projected into the UMAP coordinates with the scv.pl.velocity_embedding_stream function for visualization.

For trajectory inference, Partition-based graph abstraction (PAGA) has been benchmarked as the best technique. PAGA is extended by velocity-inferred directionality. Here, PAGA was used to characterize connections between cell subpopulations and infer transitions between different subclusters. CellRank is a modular framework to study cellular dynamics based on Markov state modeling of multi-view single-cell data [35]. In this study, we used the CellRank’s VelocityKernel and computed a transition matrix based on RNA velocity. To make our calculations more accurate, we combine the VelocityKernel with the similarity-based ConnectivityKernel and visualize the results using the vk.plot_projection function. CytoTRACE [36] is a computational algorithm for predicting differentiation status from scRNA-seq data. CytoTRACE leverages a simple, yet robust, determinant of developmental potential—the number of detectably expressed genes per cell, or gene counts. We used CytoTRACE as a complement to the RNA velocity analysis.

We used Slingshot [37] to infer developmental differentiation trajectories in the scRNA-seq dataset. Slingshot can serve as a component in an analysis pipeline after dimensionality reduction and clustering. In our study, we used slingshot to infer the developmental trajectories of the fibroblast subsets and the macrophage-monocyte dataset. For the fibroblast dataset, we chose Mki67^+^ fibroblasts as the starting point based on the results of the PAGA and CytoTRACE analyses. For the macrophage-monocyte dataset, combining the results of PAGA analysis and CytoTRACE analysis, we set Gpnmb^+^Fabp5^+^ macrophages as the endpoint and monocytes as the start point to analyze the cell developmental trajectory.

### pySCENIC analysis

We analyzed activated regulons in different fibroblast subpopulations using SCENIC [38]. Gene–gene co-expression relationships between transcription factors (TFs) and their potential targets were inferred using the grn function and the gene regulatory network reconstruction algorithm "grnboost2". Next, the regulator activity of each cell was calculated using the aucell algorithm.

### Animal models

All experimental animal procedures were approved by the Animal Care and Use Committee of Renmin Hospital of Wuhan University, and were also in accordance with the Guidelines for the Care and Use of Laboratory Animals published by the US National Institutes of Health. Wild-type C57BL/6 mice (male; 8–10 weeks old; 23.5–27.5 g) were purchased from the Institute of Laboratory Animal Science, Chinese Academy of Medical Sciences (Beijing, China). For MI surgery, the hearts and left anterior descending arteries (LAD) of the mice were completely exposed, and the vessels were ligated with a 7–0 suture approximately 2 m from the lower margin of the left atrial appendage under a stereomicroscope, and the distal end of the ligature appeared white due to ischemia, indicating that the procedure was successful. The mice were sacrificed on postoperative days 7 and 28 and the hearts were excised and used for subsequent studies. For TAC surgery, mice were anesthetized by intraperitoneal injection of 0.3% sodium pentobarbital (50 mg/kg), chest and axillary hairs were shaved off. The surgical region was subjected to sterilization using iodine and a 75% ethanol solution. Afterward, an incision was made in the left thoracic cavity of the mice. Subsequently, the aortic arch was ligated with a No. 7–0 silk thread and a No. 27 G needle.

### Masson’s trichrome staining

Heart tissue was fixed in 10% buffered formalin and embedded in paraffin. Transverse sections of heart tissue were cut, deparaffinised in xylene and dehydrated by the ethanol gradient method. Masson trichrome staining was performed according to the manufacturer's protocol (Servicebio, G1006, China). Finally, the sections were washed, dehydrated and sealed with a xylene-based sealer.

### Immunofluorescence staining

For fluorescent staining of heart tissue sections, paraffin-embedded sections were first de-paraffinized, rehydrated, and subjected to antigen recovery using citric acid buffer for immunofluorescent labeling of cardiac slices. For immunofluorescence staining of cell coverslips, cells were fixed with 4% paraformaldehyde for 15 min and permeabilized in 0.5% Triton X-100 for 15 min. After blocking the non-specific binding with 10% goat serum, cardiac slices or cell coverslips were incubated with the primary antibodies (Table S3) at 4 °C overnight, and stained with the goat anti-mouse IgG Alexa Fluor 488 or goat anti-rabbit IgG Alexa Fluor 568 secondary antibodies (1:100 dilution) at 37 °C for an additional 1 h. The nuclei were visualized with SlowFade^™^ gold antifade reagent with DAPI (#S36939, Invitrogen). The immunofluorescent images were obtained by a DP74 fluorescence microscope (OLYMPUS, Tokyo, Japan) and quantified with ImageJ software.

### Cardiac fibroblast isolation, culture and adenovirus infection

The cardiac fibroblasts were extracted from 3-day-old Sprague–Dawley rats. After removal of the atria and right ventricle and rinsing with pre-cooled D-Hanks solution, cardiac tissues were minced in DMEM/F12 (#51445C, Gibco) culture medium and digested with 0.125% trypsin (#25,200, Gibco) five times, each time for 10 min. The digest was then collected and centrifuged to remove the supernatant. The cells were resuspended in DMEM/F12 culture medium containing 15% FBS, filtered through a 40 µm filter and inoculated into 10 cm dishes for 90 min. Cardiac fibroblasts were incubated by adding shCtrl or shCthrc1 adenovirus to DMEM/F12 culture medium containing 10% FBS. After 12 h of incubation, the culture was switched to DMEM/F12 medium containing 10% FBS and Ang II (1 μmol/l) without adenovirus and continued for 48 h.

### RNA isolation and quantitative real-time PCR

Total RNA was extracted from the cardiac fibroblasts with TRIzol lysis reagent (#15,596,018, Invitrogen), extracted by trichloromethane, precipitated by isopropanol and rinsed by 75% ethanol. The purity as well as the concentration of the proposed RNA was determined, cDNA synthesis was performed with a Transcriptor First Strand cDNA Synthesis Kit (Roche, Basel, Switzerland). Light Cycler 480 SYBR Green 1 Master Mix was used to perform qRT-PCR. The details about all primer sequences are listed in Table S4, and GAPDH is used as the endogenous reference gene.

### Statistical analysis

Results are expressed as the mean ± SEM. Comparisons between two groups with a normal distribution and homogeneity of variance were performed using an unpaired Student’s t test. GraphPad Prism (version 9.0, GraphPad Software, San Diego, CA) was used for statistical analysis. A *P* value less than 0.05 was considered statistically significant.

## Results

### ECM-producing fibroblasts have similar phenotypes across mouse and human single-cell transcriptome atlas

**Fig. 1:**
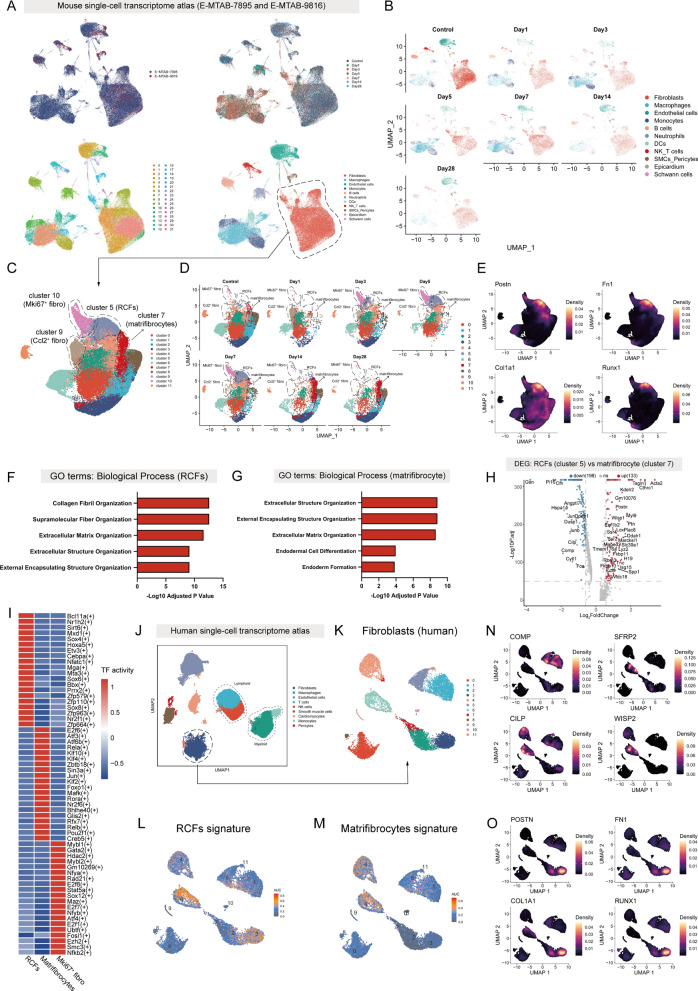
Activated fibroblasts have similar phenotypes across mouse and human single-cell data. **A** Overview of the mouse scRNA-seq datasets. The project distribution, time distribution, clusters distribution and cell types distribution are depicted as UMAP plots. **B** UMAP plots of cells from 0, 1, 3, 5, 7, 14, and 28 days after MI in the integrated mouse scRNA-seq dataset colored by cell type. **C** UMAP scRNA-seq plot of fibroblasts from the integrated mouse scRNA-seq dataset. A total of 12 clusters were identified. **D** UMAP plots of fibroblasts from 0, 1, 3, 7, 14, and 28 days after MI colored by cluster. **E** Gene expression of Postn, Fn1, Col1a1 and Runx1. **F** GO enrichment analysis of the top 50 genes in cluster 5 (RCFs, mouse). **G** GO enrichment analysis of the top 50 genes in cluster 7 (matrifibrocyte, mouse).** H** The volcano plots showing the DEGs between cluster 5 vs. cluster 7. **I** Heatmap showing TFs activity for cluster 5, cluster 7 and cluster 10 fibroblasts subsets (mouse).** J** UMAP plot of cells from the integrated human scRNA-seq dataset colored by cell type. **K** UMAP scRNA-seq plot of fibroblasts from the integrated human scRNA-seq dataset. A total of 12 clusters were identified. **L** UMAP plots showing mouse RCFs scRNA-seq signatures using the top 10 DEGs from mouse RCF mapped into fibroblasts from the integrated human scRNA-seq dataset. **M** UMAP plots showing mouse cluster 7 scRNA-seq signatures using the top 10 DEGs from mouse cluster 7 mapped into fibroblasts from the integrated human scRNA-seq dataset. **N** Gene expression of COMP, SFRP2, CILP and WISP2. **O** Gene expression of POSTN, FN1, COL1A1 and RUNX1

To comprehensively characterize the subpopulations of fibroblasts in MI, we integrated two previously published scRNA-seq datasets [16,19]. After quality control and filtering, we ended up with a dataset containing 12,0121 cells categorized into 11 different cell types (Fig. 1A). These cells were extracted from mouse hearts 1, 3, 5, 7, 14 and 28 days after MI (Fig. 1B). We extracted fibroblasts from the integrated scRNA-seq dataset and re-clustered them to get higher resolution for heterogeneity and ultimately identified 12 subclusters (Fig. 1C). Analyzing the composition of fibroblasts at different time points we identified three subsets of fibroblasts (cluster 5, cluster 9 and cluster 10) that were absent in controls but appeared during the acute phase of the MI period (Fig. 1D). Next, we analyzed all the fibroblast subsets to identify genes characteristic of different subsets (Table S5). Characterization genes for cluster 5 included Cthrc1, Acta2, Fn1, Col1a1 and Postn (Figure S1A; Table S5). Characterization genes for cluster 9 included Mt2, Ccl2, Timp1, Mt1 and Prg4. Cluster 9 was present in large numbers on day 1 after MI, decreases rapidly on day 5, and exhibited high expression of genes related to immunity. Characterization genes for cluster 10 included Stmn1, H2afz, Cks2, Cenpa and Acta2 (Figure S1A; Table S5). We also found that MKi67 was almost exclusively expressed in cluster 10 (Figure S1B), suggesting that cluster 10 represents a subset of fibroblasts with high proliferative capacity. Of note, we identified cluster 7, which is abundant in the late phase of MI but rare in the control and acute phases of MI. Characterization genes for cluster 7 included Comp, Sfrp2, Cilp, Eln, Wisp2 and Ctgf (Figure S1A; Table S5). This subcluster exhibits the characteristic of matrifibrocytes, which appeared late in the infarction phase as mentioned in the previous study, persisted in the scarred area, expressed the Comp gene, and lost αSMA expression (Figure S1A) [39,40]. The biological processes of cluster 5 were enriched in the collagen fibril organization and ECM organization (Fig. 1F), suggesting that this cluster was closely associated with fibrotic scar formation and may represent the reparative cardiac fibroblasts (RCFs) mentioned in a previous study [23]. To test our hypothesis, we reanalyzed the Ruiz-Villalba et al. generated scRNA-seq, and clustering analysis found Cthrc1 is highly expressed in cluster 3, suggesting that cluster 3 represents the RCFs mentioned by Ruiz-Villalba et al. (Figure S1C and S1D). We then extracted the top 50 DEGs in this subpopulation and mapped them to our fibroblast dataset, and finally found this signature was highly expressed in cluster 5, in concordance with our hypothesis (Figure S1E). Of note, we found that the top 50 DEGs of matrifibrocytes, which appeared in the late phase of MI, were also predominantly enriched with ECM organization (Fig. 1G). DEGs analysis further confirmed that Postn, Cthrc1 and Acta2 were expressed in RCFs, while Comp and Gsn were enriched in matrifibrocytes (Fig. 1H). We next used pySCENIC [38] to explore whether the gene regulatory structure (regulons) in RCFs was deranged compared with the matrifibrocytes. The results showed significant differences in the TFs of these two types of fibroblasts, with RCFs exhibiting much higher expression of transcription factors such as Bcl11a and Sirt6, as well as high expression of SOX4, SOX6 and SOX8 (Fig. 1I). Among them, SOX6 is considered an important target for cardiovascular disease [41]. Matrifibrocytes, in contrast, had high expression of E2f6 and Atf3 (Fig. 1I). GO enrichment analysis of Mki67^+^ fibroblasts showed that the DEGs were mainly enriched in mitotic spindle organization, microtubule cytoskeleton organization involved in mitosis and mitotic cell cycle phase transition (Figure S1F). DEGs analysis showed that Col1a1, Col3a1 and Sparc were expressed in RCFs, while Stmn1 and Cenpa were enriched in Mki67^+^ fibroblasts (Figure S1G).

To compare fibroblast subsets in human heart tissues, we downloaded snRNA-seq datasets of MI and ICM from the Human Cell Atlas and the GEO database and performed quality control and integration and a dataset of 72,515 cells was finally obtained. Clusters were annotated with marker genes from the literature [12,15] and nine major cardiac cell types were identified (Fig. 1J). we extracted fibroblasts from the human scRNA-seq dataset and reclustered them for analysis. Unbiased clustering eventually grouped cardiac fibroblasts into 12 different subsets (Fig. 1K). We first mapped the gene expression signature of mouse RCFs using an AUC score. This signature was most highly expressed in human cluster 3 and cluster 6 (Fig. 1L). At the same time, the marker gene (POSTN, FN1, COL1A1 and RUNX1), which was highly expressed in mouse RCFs, was also highly expressed in human fibroblasts cluster 3 (Fig. 1O). This result suggests that human fibroblasts cluster 3 and mouse RCFs have similar phenotypes. Next, we mapped the gene expression signature of mouse matrifibrocytes using an AUC score. This signature was highly expressed in human cluster 6, cluster 4 and some cells in cluster 1 and cluster 5 (Fig. 1M). SFRP2, CILP and WISP2 were highly expressed in cluster 6, whereas COMP was mainly expressed in cluster 1 and cluster 5 (Fig. 1N). We simultaneously analyzed human cluster 3 (human RCFs) for DEGs analysis with human cluster 6 and human cluster 0, respectively (Figure S1H and S1I). GO enrichment analysis of human cluster 3 showed that the DEGs were mainly enriched in collagen fibril organization and extracellular matrix organization, while the DEGs in human cluster 6 were mainly enriched in cytoplasmic translation and peptide biosynthetic process (Figure S1J and S1K). In summary, by dynamically observing the changes in fibroblast subsets after MI, we observed four subsets of fibroblasts appearing after infarction, including Mki67^+^ fibroblasts, RCFs, Ccl2^+^ fibroblasts and matrifibrocytes. The signature of ECM-producing myofibroblasts (RCFs and matrifibrocytes) were also present in human cardiac single-cell transcriptome atlas.

### Mki67^+^ fibroblasts contribute to RCFs during the acute phase of MI

**Fig. 2:**
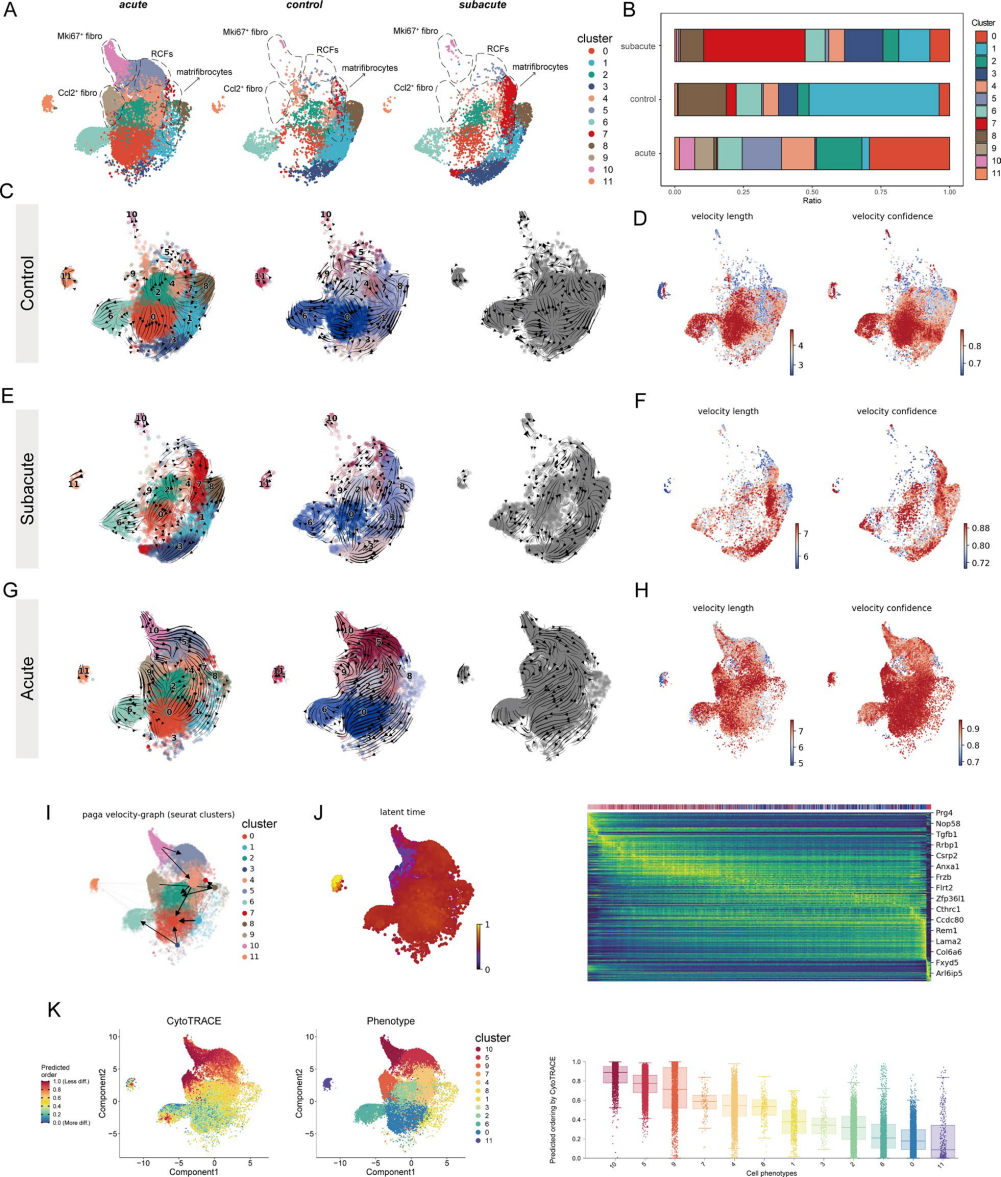
Mki67^+^ fibroblasts contribute to RCFs during the acute phase of MI. **A** UMAP plots of fibroblast clusters showing time distribution. **B** Proportion of fibroblast clusters showing in bar plots from control, stable and acute groups. **C** RNA velocity analysis showing the transition potential among fibroblast clusters at the control group, transcription dynamics based on the dynamical model (left), transcription dynamics based on the stochastic model (middle). Using CellRank’s VelocityKernel to compute transition matrix based on RNA velocity (right). **D** The velocity length (left) and velocity confidence (right) of the fibroblasts in control group. **E** RNA velocity analysis showing the transition potential among fibroblast clusters at subacute group, transcription dynamics based on dynamical model (left), transcription dynamics based on stochastic model (middle). Using CellRank's VelocityKernel to compute transition matrix based on RNA velocity (right). **F** The velocity length (left) and velocity confidence (right) of the fibroblasts in subacute group. **G** RNA velocity analysis showing the transition potential among fibroblast clusters at the acute group, transcription dynamics based on the dynamical model (left), transcription dynamics based on the stochastic model (middle). Using CellRank's VelocityKernel to compute transition matrix based on RNA velocity (right). **H** The velocity length (left) and velocity confidence (right) of the fibroblasts in acute group. **I** UMAP analysis of all fibroblast clusters at acute group embedded with PAGA connectivities for trajectory inference.** J** UMAP plots colored by the recovered latent time in the scRNA-seq datasets at acute group (left) and expression heatmaps for top 100 genes with fibroblasts ordered by latent time values. **K** UMAP plots of differentiation levels and the distribution of fibroblasts clusters (left). Boxplot showing the comparison of CytoTRACE score between different fibroblasts clusters (right)

To explore the differentiation trajectories of different subsets of fibroblasts during MI, we utilized the E-MTAB-7895 dataset for RNA velocity analysis since this method requires raw sequencing data. We divided these fibroblasts into three groups based on different periods: acute phase of MI (1, 3, 5, 7 days post-MI), subacute phase of MI (14, 28 days post-MI), and control group (non-surgical) (Fig. 2A and 2B). Here, we used two different models “stochastic” and “dynamical” to estimate RNA velocity, and we also used CellRank to compute a transition matrix based on RNA velocity. We calculated the rate of cell differentiation, which can be measured by velocity length. We first analyzed the fibroblast populations in the subacute and control phases. We found that cluster 0 in the control group may contribute to other fibroblast subsets, with no clear evolutionary sequence between the various subsets (Fig. 2C). This is consistent with the higher rate of differentiation of cluster 0 (Fig. 2D). Similarly, there was no significant differentiation trajectory at 14 and 28 days after MI (Fig. 2E). Of note, cluster 7 (matrifibrocytes), a subset of fibroblasts appearing mainly in the subacute group exhibited a higher rate of differentiation (Fig. 2F). In sum, our RNA velocity analysis results showed that the transitions between fibroblast subsets in the later stages of MI resembling the dynamics between fibroblast subsets observed in healthy myocardium.

We next focused on RNA velocity analysis of fibroblast subsets in the acute phase of MI. We found that the results of the RNA velocity analysis of the “stochastic” model were similar to those of the “dynamical” model and the transition matrix based on RNA velocity. In the period from 1 to 7 after MI, we found a transition from cluster 10 (Mki67 + fibroblasts) to cluster 5 (RCFs), immediately followed by a transition from RCFs to matrifibrocytes, cluster 4 (Fig. 2G). This is consistent with a previous study that matrifibrocytes are transformed from myofibroblasts [39]. We also observed a transition from cluster 9 to cluster 0 and cluster 2. Cluster 0, cluster 1 and cluster 3 are supposed to be the terminal subgroups of differentiation. Whereas there were no significant differences in differentiation rates between subsets of fibroblasts in the acute group (Fig. 2H). PAGA of RNA velocity provides a graph-like map of the data topology with weighted edges corresponding to the connectivity between two clusters [42]. The PAGA velocity graph also showed a direction from Mki67^+^ fibroblasts to RCFs (Fig. 2I). The above results suggested that Mki67^+^ fibroblasts, which were rapidly activated after MI injury, were converted to RCFs and participated in subsequent cardiac wound healing. However, latent time analysis showed no significant difference between RCFs and Mki67^+^ fibroblasts (Fig. 2J). We also used CytoTRACE to assess the differentiation potential of different fibroblast subsets and obtained similar results to the RNA rate analysis, with cluster 10 (Mki67^+^ fibroblasts), cluster 5 (RCFs), cluster 9 (Ccl2^+^ fibroblasts) and cluster 7 (matrifibrocytes) being the subsets with the highest differentiation capacity, representing an intermediate differentiation state (Fig. 2K). Finally, we used Slingshot to track the differentiation trajectory of fibroblast subsets. We set Mki67^+^ fibroblasts as our starting point because Mki67^+^ fibroblasts have a higher differentiation capacity compared to other subsets. Consistent with the results of the above analysis, RCFs were derived from Mki67^+^ fibroblasts and served as an intermediate for the eventual emergence of other fibroblast subsets (Figure S2A through S2E).

### CTHRC1^+^ fibroblasts represent an activated fibroblast population in chronic disease state

**Fig. 3:**
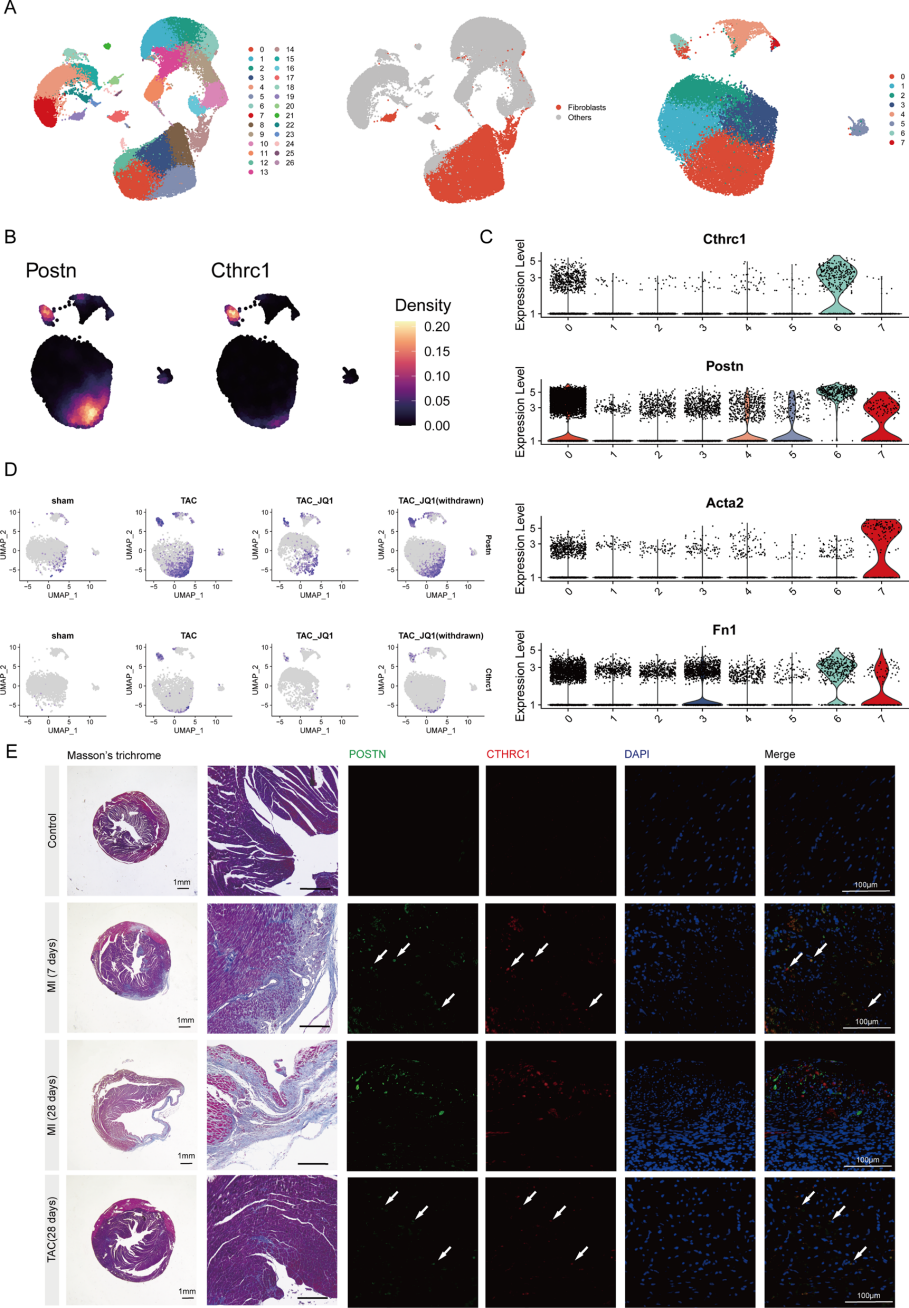
Characterization of CTHRC1^+^ fibroblasts in a mouse model of heart failure. **A** UMAP plot showing the fibroblast and their subclusters. A total of 8 clusters were identified. All other types of cells were colored in gray. **B** Gene expression of Postn and Cthrc1. **C** Expression by cluster of known activated fibroblast related genes shown as violin plots in fibroblasts. **D** Expression by sample of Postn and Cthrc1 shown as feature plots in fibroblasts. **E** Spatial location of POSTN, CTHRC1 in the control group, the infarct zone at 7, 28 days post MI and 28 days post TAC. POSTN (green), CTHRC1 (red), Nuclei (DAPI, blue). Co-localizations are in yellow (arrows). Scale bars: 100 µm

Our results indicate that Cthrc1 was the top marker gene in RCFs, but RCFs were predominantly seen in the acute phase after MI injury. Previous studies have shown that CTHRC1 also appears in the late infarct stage and is expressed in human cardiac tissues from ICM and DCM patients [23]. To further explore the gene expression level of CTHRC1 in cardiac fibrosis and its correlation with the chronic disease state, we obtained the scRNA-seq datasets GSE155882 and GSE185265 related to cardiac fibrosis in mice from the GEO database. The GSE155882 dataset includes mice treated in four different subgroups, while GSE185265 contains three subgroups (Figure S3A). We first integrated the data from different treatment groups in GSE155882 and then extracted fibroblast subpopulations based on the marker gene of fibroblasts and performed dimensionality reduction clustering analysis (Fig. 3A). Fibroblast populations with high expression of Cthrc1 also had high expression of Postn, a classical marker of activated fibroblasts (Fig. 3B). DEGs analysis revealed a significant upregulation of Postn and Cthrc1 in cluster 6 (Fig. 3C). The expression of Postn was reduced in fibroblasts after treatment with JQ1, a drug that effectively reduces fibrosis and improves cardiac function [22], and this effect was attenuated after the withdrawal of JQ1 midway. Similar to the results for Postn, Cthrc1 expression in fibroblasts was reduced after JQ1 treatment (Fig. 3D). We used the same approach to integrate and process another scRNA-seq dataset GSE185265 (Figure S3B). Similar to the results of the TAC model, Cthrc1^+^ fibroblasts highly express Postn (Figure S3C). The expression level of Cthrc1 was significantly reduced in the late stage of MI, but it was still evident that Postn and Cthrc1 expression levels were reduced in TTg mice [21] (better heart function and less cardiac fibrosis) compared to control mice (Figure S3D and S3E). Our immunofluorescence results showed that some Postn-positive fibroblasts also expressed Cthrc1 at 7 days after MI and in TAC model hearts, but such fibroblasts were rarely present in cardiac tissues in the control group and at 28 days after MI (Fig. 3E). Taken together, the analysis of the two mouse scRNA-seq datasets suggested that in chronic disease states, fibroblast populations with high Cthrc1 expression (Cthrc1^+^ fibroblasts), a subset of Postn^+^ fibroblasts, are associated with worsened cardiac function and exacerbated fibrosis.

To explore whether there are similar results in human heart tissue, we collected snRNA-seq datasets of human DCM and HCM (Figure S4A). We first integrated multiple DCM samples and then extracted fibroblasts which were finally divided into 5 subsets (Figure S4B). Compared with other subsets, cluster 5 and cluster 6 highly expressed POSTN, and were mainly present in the DCM group (Figure S4C and S4D). In addition, we found that CTHRC1 was mainly expressed in cluster 5 and highly overlapped with POSTN^+^ fibroblasts. For HCM, unbiased clustering grouped the fibroblasts into six clusters. Cluster 1 and cluster 5 increased considerably in HCM vs. CTRL (Figure S4F and S4G). Consistent with this, cluster 1 and cluster 5 were highly expressive of genes associated with activated fibroblasts such as POSTN and MEOX1 [22], meanwhile CTHRC1 was also predominantly expressed in relation to these two subsets (Figure S4H). Compared to controls, fibroblasts from the HCM group exhibited higher expression of CTHRC1 (Figure S4I). The results of GO enrichment analysis for cluster 1 and cluster 5 also showed that the DEGs were mainly enriched in the ECM organization, extracellular structure organization and collagen fibril organization (Figure S4J). Similar to the results obtained from the mouse scRNA-seq dataset, CTHRC1^+^ fibroblast in DCM and HCM often also have high expression of POSTN and represents activated fibroblasts.

### Activator protein-1 is a common transcription factor in CTHRC1^+^ fibroblasts in chronic disease state

**Fig. 4:**
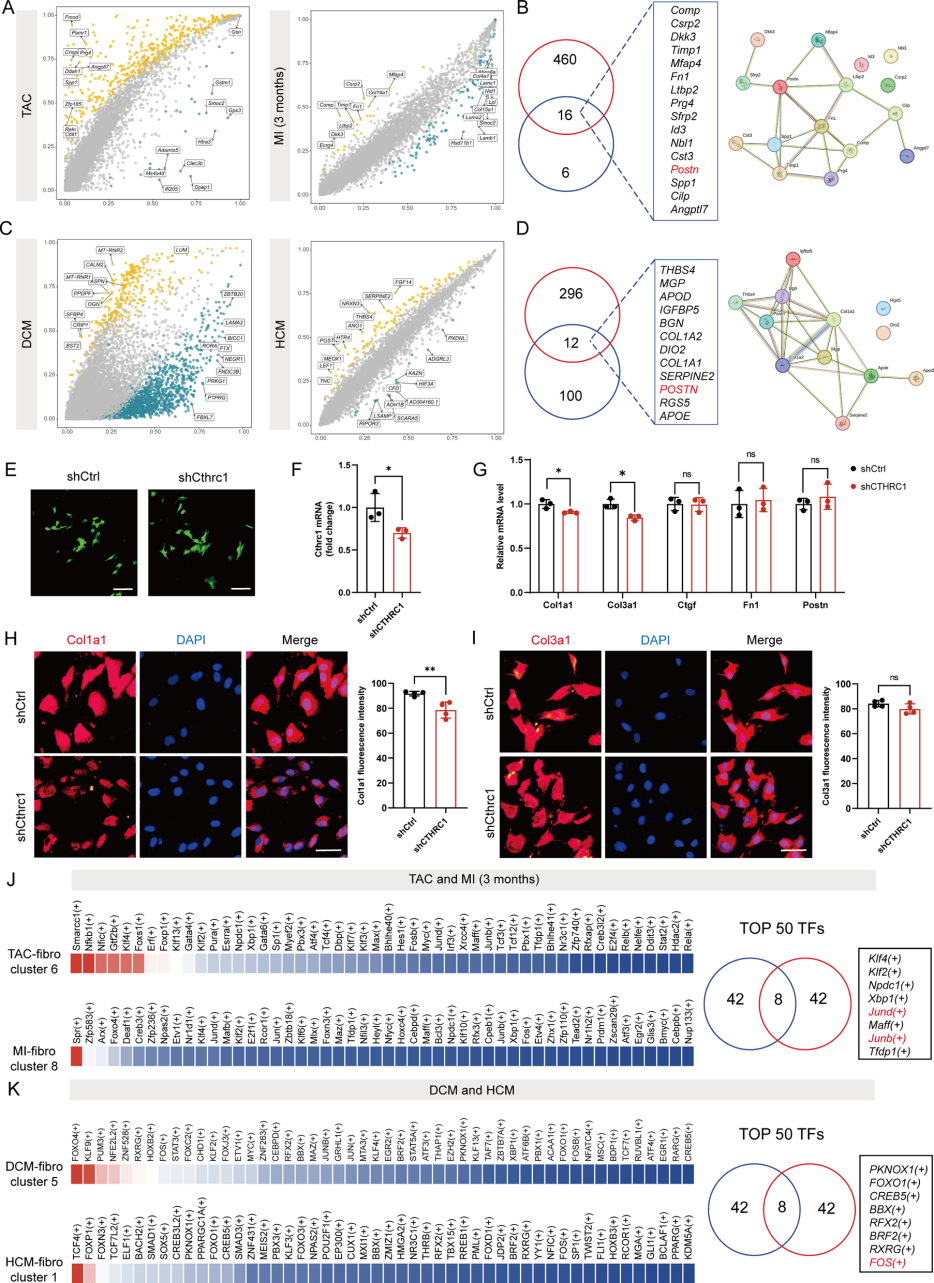
Gene expression profile of CTHRC1^+^ fibroblasts. **A** Volcano plot of DEGs in Cthrc1^+^ fibroblasts versus all other types of fibroblasts. **B** Shared differentially expressed genes and their PPI network. **C** Volcano plot of DEGs in Cthrc1^+^ fibroblasts versus all other types of fibroblasts. **D** Shared differentially expressed genes and their PPI network. **E**–**F** GFP expression level and mRNA expression level of CTHRC1 in the shCtrl and shCthrc1 groups. Scale bars: 100 µm **G** The mRNA expression level of Col1a1, Col3a1, Ctgf, Postn, Fn1 in the shCtrl and shCthrc1 groups (n = 3). **H** Col1a1 expression detected by cellular immunofluorescence staining (n = 4). Scale bars: 50 µm.** I** Col3a1 expression detected by cellular immunofluorescence staining (n = 4). Scale bars: 50 µm. **J** Heatmap showing top 50 TFs activity for TAC-fibroblast cluster 6 (Cthrc1^+^ fibroblasts) and MI-fibro cluster 8 (Cthrc1^+^ fibroblasts). Venn diagram showing TFs common to both disease models. **K** Heatmap showing top 50 TFs activity for DCM-fibroblast cluster 5 (CTHRC1^+^ fibroblasts) and HCM-fibro cluster 1 (CTHRC1^+^ fibroblasts). Venn diagram showing TFs common to DCM and HCM. *P < 0.05, **P < 0.01

To better understand the characteristics of CTHRC1^+^ fibroblasts, we subjected CTHRC1^+^ fibroblasts to DEG analysis with all other fibroblast subpopulations. We took the intersection of the genes upregulated in the dataset of TAC mice with the genes whose expression was upregulated in the dataset of MI 3 month mice, and finally obtained 16 genes (Fig. 4A and 4B). These intersecting genes included Comp, Fn1, Cilp, Spp1 and Postn, which were associated with fibrosis. Similarly, the common genes that were highly expressed in CTHRC1^+^ fibroblasts in DCM and HCM compared to other fibroblast subpopulations also included profibrotic genes such as POSTN, COL1A1 and COL1A2 (Fig. 4C and 4D). We transfected the constructed adenovirus into cardiac fibroblasts to knock down the Cthrc1 gene in the cells, and the results of qRT-PCR showed that the Cthrc1 gene was successfully knocked down after infection (Fig. 4E and 4F). We found that expression of collagen-associated mRNA levels was reduced in Cthrc1 knockdown cells under Ang II stimulation compared to shCtrl group (Fig. 4G). Similarly, immunofluorescence results showed that the fluorescence intensity level of Col1a1 was lower in the shCthrc1 group compared to the shCtrl group, but the fluorescence intensity of Col3a1 was not significantly different between the two groups (Fig. 4H and I). We next analyzed the TFs of CTHRC1^+^ fibroblasts using pySCENIC in an attempt to discover their shared transcription factor profile. Common TFs for Cthrc1^+^ fibroblasts in the TAC model and 3 months after MI included Klf4, Klf2, Npdc1, Xbp1, Jund, Maff, Junb and Tfdp1 (Fig. 4J). TFs shared by CTHRC1^+^ fibroblasts in DCM and HCM were PKNOX1, FOXO1, CREB5, BBX, RFX2, BRF2, RXRG and FOS (Fig. 4K). Combining the results of these analyses we found that activator protein-1 (such as JUND, JUNB and FOS) may be a common transcription factor in CTHRC1^+^ fibroblasts. Altogether, the above results suggested that, CTHRC1^+^ fibroblasts in the chronic disease state tend to represent persistently activated fibroblasts and activator protein-1 may be involved in the regulation of CTHRC1^+^ fibroblasts.

### Gpnmb^+^Fabp5^+^ macrophages possess characteristics of SAMs and SPP1^hi^ macrophages present in the early stages of MI

**Fig. 5:**
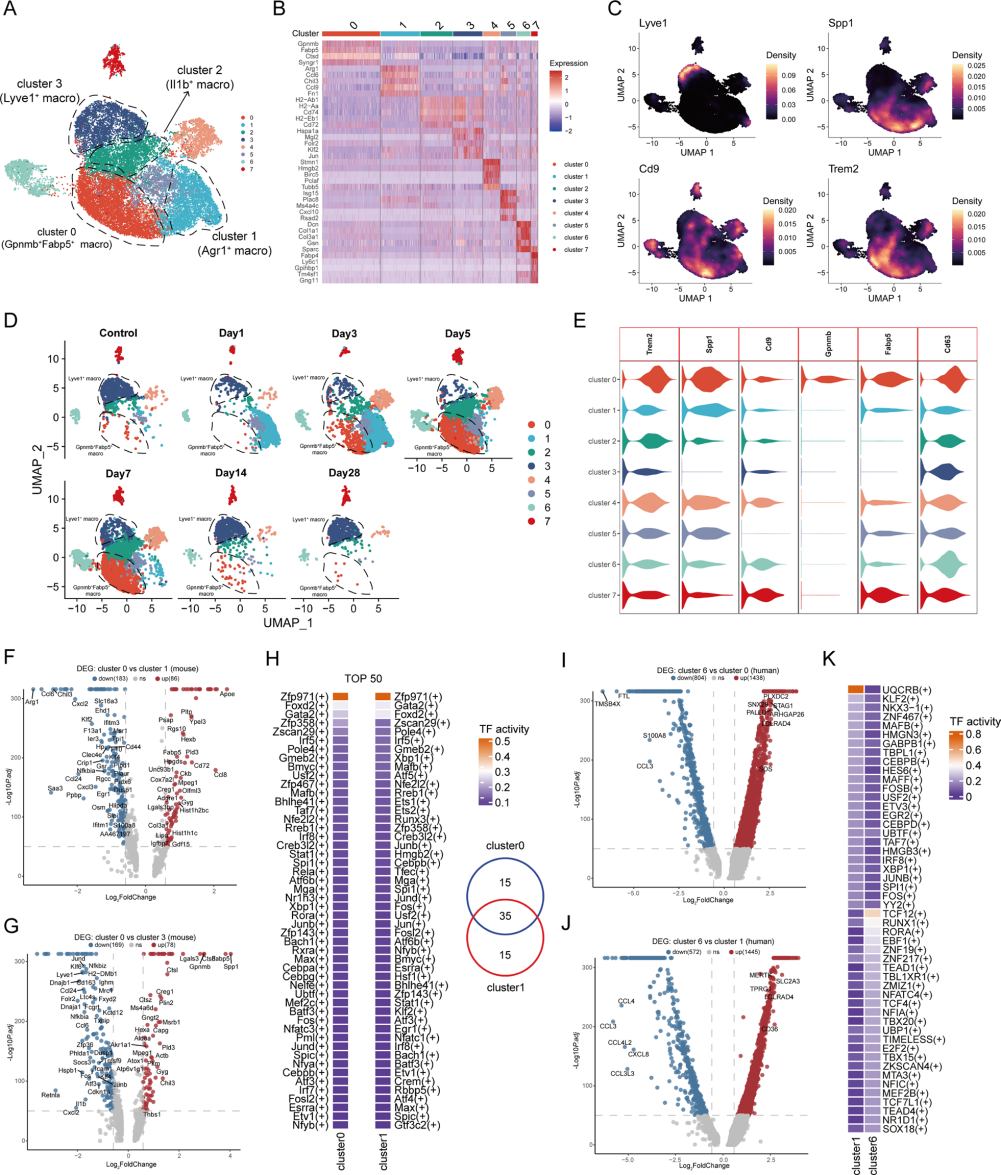
Gpnmb^+^Fabp5^+^ macrophage possess characteristics of SAMs.** A** UMAP plot of macrophages from the integrated mouse scRNA-seq dataset. A total of 8 clusters were identified. **B** Heatmap showing the expression profiles of top 5 genes ranked by LogFC of each cluster. **C** Gene expression of Lyve1, Spp1, Cd9 and Trem2. **D** UMAP plots of macrophages from 0, 1, 3, 7, 14, and 28 days after MI colored by cluster. **E** Violin plots showing the expression of Trem2, Spp1, Cd9, Gpnmb, Fabp5 and Cd63 in macrophage subsets. **F** The volcano plots showing the DEGs between cluster 0 vs. cluster 1 (mouse). **G** The volcano plots showing the DEGs between cluster 0 vs. cluster 3 (mouse). **H** Heatmap showing the top 50 TFs activity for cluster 0 and cluster 1 and cluster 10 macrophages subsets (mouse). Venn diagram showing TFs common to both subsets. **I** The volcano plots showing the DEGs between cluster 6 vs. cluster 0 (human). **J** The volcano plots showing the DEGs between cluster 6 vs. cluster 1 (human). **K** Heatmap showing the TFs activity for cluster 1 and cluster 6 macrophages subsets (human)

Immune-fibroblast interaction is an important mechanism of MI. To study macrophage alterations after MI, we extracted macrophages from the integrated mouse scRNA-seq dataset, and unbiased clustering grouped the macrophages into 8 subclusters (Fig. 5A and 5B). Cluster 3 high expression of Lyve1 may represent a resident Lyve1^+^ macrophage (Fig. 5C). It has recently been suggested that SAMs express TREM2 and/or CD9 in liver fibrosis and lung fibrosis [7]. We therefore validated the expression levels of these genes in different macrophage subsets. We found that Cd9 and Trem2 were expressed in several subsets including cluster 0, cluster 1, cluster 2, and cluster 4 (Fig. 5C). Spp1^+^ macrophages, which have recently been suggested to be strongly associated with tissue fibrosis [43,44]. We found that Spp1 was highly expressed in cluster 0, cluster 1 and cluster 4 (Fig. 5C). Cluster 0 and cluster 4 began to expand mainly at 3 days after MI, peaking at day 7, but decreased dramatically on day 14, whereas cluster 1 expanded rapidly on day 1 after MI but decreased substantially on day 5 (Fig. 5D). DEGs analysis of the different subsets revealed that genes highly expressed in cluster 0 included Gpnmb, Fabp5, Ctsd, Syngr1, Trem2, Cd63 and Spp1 (Fig. 5E; Table S6). In concordance with this, compared with the cluster 1 and cluster 3, cluster 0 highly expresses Apoe, Gpnmb, Lgals3 and Spp1 (Fig. 5F and 5G). The temporal sequentiality of the appearance of cluster 1 and cluster 0 and their positional proximity in the UMAP plot led us to speculate whether they share similar transcriptional features. The results of pySCENIC analysis showed that 35 of the top 50 TFs were the same between cluster 0 and cluster 1 including core transcription factors such as Spi1, Cebpb and Irf8 that are associated with macrophage lineage commitment and differentiation [45] (Fig. 5H). We also discovered cluster 0 exhibits some characteristic LAMs (lipid-associated macrophages) transcripts [46,47]. Meanwhile, the expression of cluster 0 was characterized like the previously reported SAMs, which highly express GPNMB, FABP5, SPP1 and CD63 [7]. Thus cluster 0 represents the Gpnmb^+^Fabp5^+^ macrophage possessing both LAMs and SAMs related characteristics. Considering the overlap of marker genes between LAMs and SAMs, further investigations are warranted to understand whether LAMs and SAMs represent the same subsets of macrophages in tissues.

Gpnmb^+^Fabp5^+^ macrophage was dramatically reduced in 14 days after MI, making us wonder if it plays a role mainly in the early stages after MI injury. To validate this hypothesis we additionally integrated three mouse scRNA-seq datasets that contained CD45^+^ cells isolated from the infarcted area of the heart (Figure S5A). We extracted macrophages from the integrated scRNA-seq dataset, sub-clustering of macrophages led to the identification of 10 clusters (Figure S5B). To compare macrophage subpopulations in the newly integrated scRNA-seq dataset, we constructed a gene expression signature containing the top 30 genes of the Gpnmb^+^Fabp5^+^ macrophage and then mapped it to the new macrophage dataset. This signature was highly expressed in cluster 2 (Figure S5C). Similar to previous results, cluster 2 appeared mainly in the 3 to 7 days period of MI and decreased significantly in the later stages (14 days post MI) (Figure S5D). At the same time, compared to other macrophage subpopulations cluster 2 highly expressed Spp1 and Gpnmb (Figure S5E). These results suggested that Gpnmb^+^Fabp5^+^ macrophage expands rapidly during the acute phase of MI and disappears in the later stages of the disease.

To explore whether Gpnmb^+^Fabp5^+^ macrophage transcriptional signature was conserved in the human diseased heart, we extracted macrophages from the above integrated human scRNA-seq dataset and performed unbiased clustering (Figure S6A). Cluster 0 high expression of LYVE1 represented cardiac resident macrophages, and the SPP1 was mainly expressed in cluster 6 (Figure S6B). CD9 and TREM2 were highly expressed in cluster 1 (Figure S6B). We then mapped the Gpnmb^+^Fabp5^+^ macrophage signature to the human macrophage dataset and found that these features were mainly expressed in cluster 1 and cluster 0 (Figure S6C). At the same time, we found that CD63 and FABP5 were also predominantly expressed in these two clusters (Figure S6D). Interestingly, we found that SPP1 was predominantly expressed in cluster 6, but not in cluster 1 and cluster 0, which is not consistent with the expression profile in the mouse dataset. We then divided the cells into three different groups based on their origin: control, MI and ICM. Cluster 6 (SPP1^hi^ macrophages) were mainly present in the MI group, cluster 0 (LYVE1^hi^ macrophages) was predominantly found in healthy cardiac tissue, whereas cluster 1 (CD9^hi^TREM^hi^ macrophages) was predominantly found in ICM patient cardiac tissue. (Figure S6E). GO enrichment analysis of cluster 6 showed that the DEGs were mainly enriched in the regulation of cell migration, extracellular structure organization and external encapsulating structure organization (Figure S6F). The gene expression profiles between cluster 6 and cluster 1, and between cluster 6 and cluster 0 were highly differentiated (Fig. 5I, J). In concordance with this, we found that the TFs were also very different between cluster 6 and cluster 1 (Fig. 5K). Overall, the results of our analyses indicated that, unlike the mouse dataset, macrophages possessing the Gpnmb^+^Fabp5^+^ macrophage signature in the human dataset were mainly present in the ICM but not in the MI cardiac tissue. However, in both mouse and human cardiac tissues, macrophages that highly express SPP1 (SPP1^hi^ macrophages) were almost exclusively present in the early stages of MI, not in the later stages of the disease (post-MI > 14 days or ICM group).

### Dynamic transition of macrophage subsets in the acute phase of MI

**Fig. 6:**
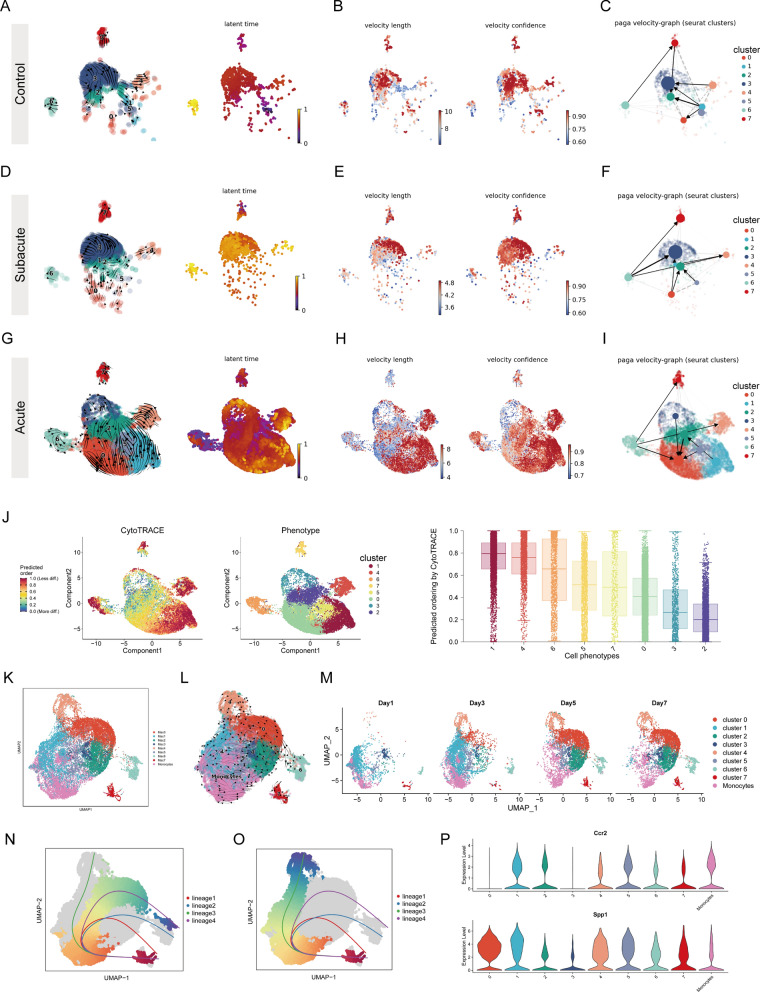
Dynamic transition of macrophage subsets in acute phase of MI. **A** UMAP plots of RNA velocity results (left) and the recovered latent time (right) in the single cell data sets at control group. **B** The velocity length (left) and velocity confidence (right) of the macrophages at control group. **C** UMAP analysis of all macrophages clusters at control group embedded with PAGA connectivities for trajectory inference. **D** UMAP plots of RNA velocity results (left) and the recovered latent time (right) in the single cell data sets at subacute group. **E** The velocity length (left) and velocity confidence (right) of the macrophages at subacute group. **F** UMAP analysis of all macrophages clusters at subacute group embedded with PAGA connectivities for trajectory inference. **G** UMAP plots of RNA velocity results (left) and the recovered latent time (right) in the single cell data sets at acute group. **H** The velocity length (left) and velocity confidence (right) of the macrophages at acute group. **I** UMAP analysis of all macrophages clusters at acute group embedded with PAGA connectivities for trajectory inference. **J** UMAP plots of differentiation levels and the distribution of macrophages clusters (left). Boxplot showing the comparison of CytoTRACE score between different macrophages clusters (right). **K** UMAP plots of cardiac macrophages and monocytes in acute stage, macrophages contain 8 clusters. **L** RNA velocity analysis showing the transition potential among macrophages clusters and monocytes. **M** UMAP plots of macrophages clusters and monocytes showing time distribution.** N** Slingshot differentiation trajectory analyses of macrophages-monocytes in the lineage 4. **O** Slingshot differentiation trajectory analyses of macrophages-monocytes in the lineage 3. **P** Violin plots showing the expression of Ccr2 and Spp1 in the macrophages-monocytes dataset

We introduced RNA velocity analysis to profile the dynamics of different subpopulations of mouse macrophages at different stages after MI injury. RNA velocity analysis showed that the majority of macrophage subpopulations in the control group flowed toward cluster 2 and cluster 3 (Fig. 6A). The PAGA plot abstraction obtained similar results, with both cluster 4 and cluster 1 shifting toward cluster 3, and cluster 0 and cluster 1 shifting toward cluster 2 (Fig. 6C). The dynamic flow of macrophages during the subacute phase was similar to the results of the control group (Fig. 6D). The results of the PAGA plot abstraction show that cluster 6 and cluster 0 flow to cluster 3, and cluster 0 and cluster 5 flow to cluster 2 (Fig. 6F). In both the control and subacute groups, cluster 1 showed a higher rate of differentiation (Fig. 6B and 6E). Next, we focused on analyzing the dynamics of macrophages in the acute phase, which possesses a larger number of macrophages. We use the generalized dynamical model to understand the transcriptional dynamics of macrophages. RNA velocity predicted that cluster 0, cluster 5 and cluster 1 may be an intermediate state in macrophage dynamics (Fig. 6G). This is consistent with our above observation that these subpopulations appeared in the acute phase but disappeared in the subacute phase. The results of velocity length and velocity confidence suggested that cluster 1 has a faster speed of differentiation compared to other macrophage subpopulations, which was consistent with the fact that cluster 1 presented in large numbers on the first day after infarction but then decreased dramatically on the fifth day (Fig. 6H). The PAGA velocity graph also showed the transition from cluster 2, cluster 3, cluster 5 and cluster 6 to cluster 0, suggesting that at the acute stage cluster 0 represents a terminally differentiated subpopulation (Fig. 6I). We also used CytoTRACE to assess the differentiation capacity of different macrophage subpopulations, and similar to the results of our RNA velocity analyses, cluster 1 had a high differentiation capacity and represented a rapidly increasing population after MI (Fig. 6J).

To explore whether cluster 0 (Gpnmb^+^Fabp5^+^ macrophages) originated from monocytes, we integrated macrophages and monocytes into one dataset for analysis (Fig. 6K). RNA velocity analysis did not give a definitive indication of the direction of cellular differentiation (Fig. 6L), and we subsequently observed cellular composition at different time points and found that Gpnmb^+^Fabp5^+^ macrophages appeared on days 5–7 post-MI, whereas most monocytes and cluster 1 macrophages appeared on days 1–3 post-MI (Fig. 6M). We then used Slingshot to infer the differentiation trajectory. Combining the results of the PAGA analysis and the time point at which Gpnmb^+^Fabp5^+^ macrophages appeared, we set it as the end point of the trajectory and set the monocyte as the start point. The results of Slingshot analysis showed four different differentiation trajectories. Lineage 3 depicts a trajectory from monocytes to cluster 1 and finally to cluster 4, whereas lineage 4 depicts a trajectory from monocytes to cluster 1 and later to cluster 0 (Fig. 6N and 6O). This result suggested that Gpnmb^+^Fabp5^+^ macrophages may originate from monocytes. Meanwhile, we observed a decrease in the expression of Ccr2 and an increase in the expression of Spp1 during the differentiation from monocytes to Gpnmb^+^Fabp5^+^ macrophages, which is consistent with previous studies (Fig. 6P) [47].

### Analysis of cell–cell communication reveals an important role for TGFB1, TNF and IL1B signaling in macrophage-fibroblast interactions

**Fig. 7:**
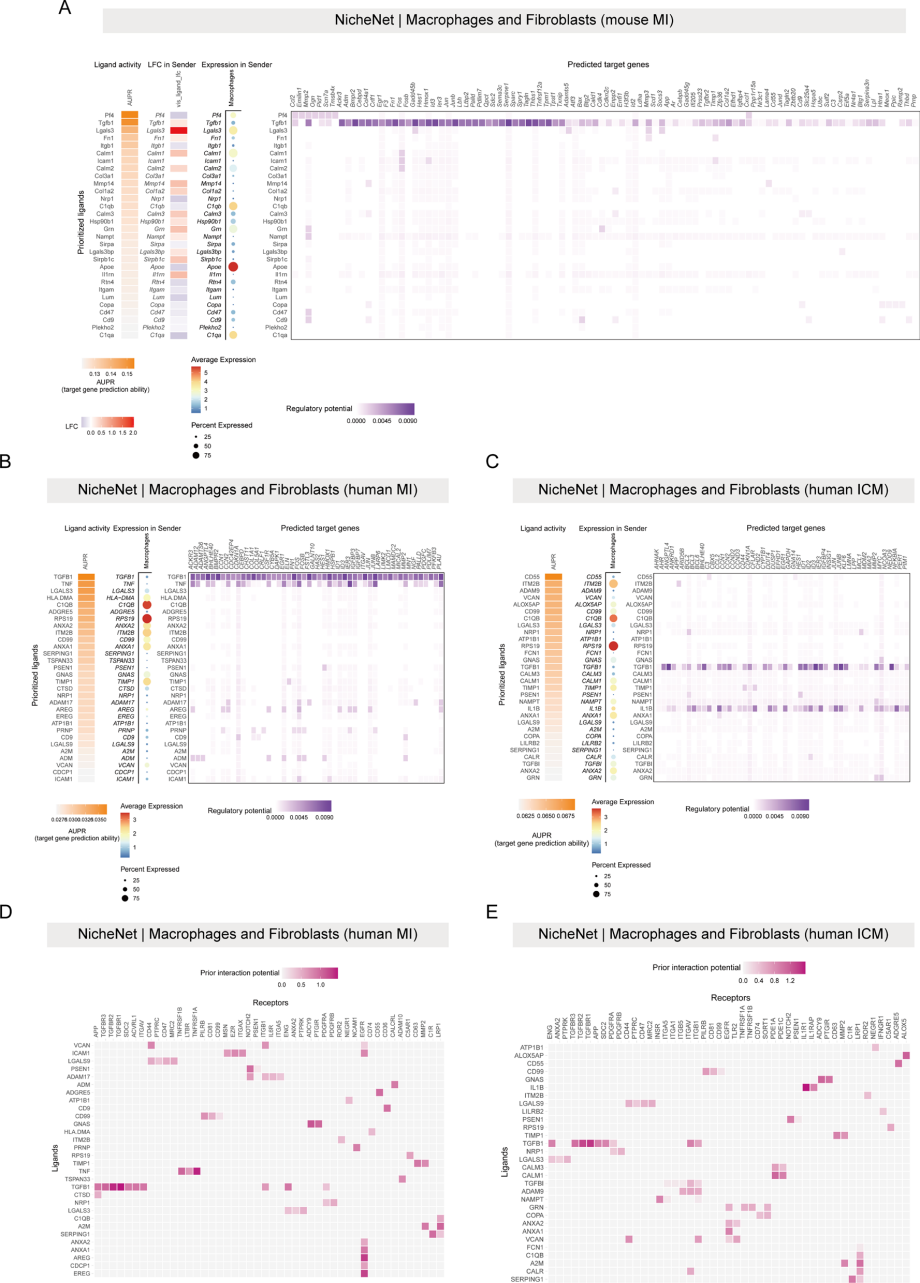
NicheNet analysis of communication between macrophages and fibroblasts. **A** Heatmaps (NicheNet) of ligand activity of top-ranking ligands expressed by macrophages (left) and their regulatory potential on predicted target genes expressed by fibroblasts (right). **B** Heatmaps (NicheNet) of ligand activity of top-ranking ligands expressed by macrophages (left) and their regulatory potential on predicted target genes expressed by fibroblasts (right). **C** Heatmaps (NicheNet) of ligand activity of top-ranking ligands expressed by macrophages (left) and their regulatory potential on predicted target genes expressed by fibroblasts (right). **D** Heatmaps of ligands expressed by macrophages and their potential receptors expressed by fibroblasts. **E** Heatmaps of ligands expressed by macrophages and their potential receptors expressed by fibroblasts

To better understand the interactions between fibroblasts and macrophages in MI and ICM, we performed NicheNet analysis, which allowed us to predict cellular interactions by linking ligand and target gene expression. We first performed the analysis using a scRNA-seq dataset of mouse divided into control and MI groups, and the DEGs in fibroblasts between the two groups were considered as the gene set of interest. Consistent with some previous findings [13,48], we found that Tgfb1 has an important role in fibrosis, as evidenced by its top-ranked predictive power (Fig. 7A). It is important to note that Pf4 also has a high predictive capacity and may interact with fibroblasts through the Pf4-Cxcl1 and Pf4-Ccl2 axes. A recent study suggested that Pf4 is a pro-youth factor that reduces age-related neuroinflammation [49]. Next, we used the human dataset for analysis, which we categorized by disease into two groups: MI and ICM. For MI, the results of the NicheNet analysis were similar to the mouse dataset, with TGFB1 ranking the top in terms of the interaction counts between macrophages and fibroblasts (Fig. 7B). At the same time, we found that TNF had a high degree of rankedness, and NicheNet analysis predicted that macrophages-derived TNF could activate JUNB and PLAU in fibroblasts (Fig. 7B). We next analyzed the possible ligands of macrophages and fibroblasts in ICM and their target genes. In addition to TGFB, macrophage-derived IL1B also showed higher association with CCL2, JUNB, and NFKBIA in fibroblasts (Fig. 7C). We also analyzed the receptors of top-ranked ligands between macrophages and fibroblasts, TGFB1-TGFBR1, TGFB1-TGFBR2, TNF-TNFRSF1A, AREG-EGFR, and EREG-EGFR have high interaction possibilities between macrophages and fibroblasts in MI pathologies (Fig. 7D). Whereas in ICM state, TGFB1-TGFBR1, TGFB1-TGFBR2, IL1B-IL1R1, CD55-ADGRE5 and ALOX5AP-ALOX5 had higher interaction potential between macrophages and fibroblasts (Fig. 7E).

### Cross-talk analysis between subclusters reveals that SPP1^hi^ macrophages interact with RCFs to promote scar formation during human MI

**Fig. 8:**
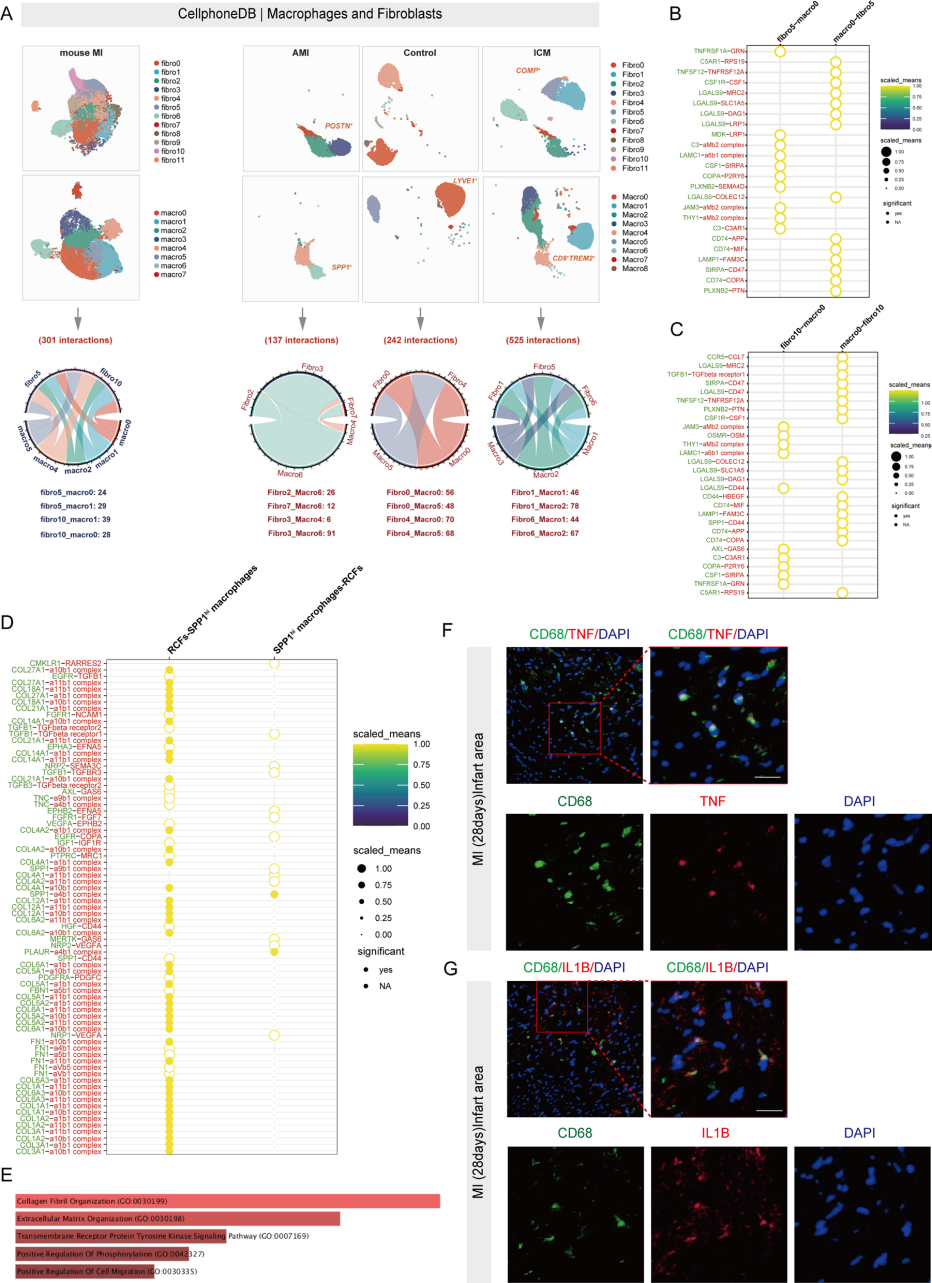
SPP1^hi^ macrophages interact with RCFs to promote scar formation during MI. **A** CellphoneDB analysis showing the interaction between macrophage clusters and fibroblast clusters. **B** Dot plot showing the ligand-receptor pairs among macro0 and fibro5 (mouse). **C** Dot plot showing the ligand-receptor pairs among macro0 and fibro10 (mouse).** D** Dot plot showing the ligand-receptor pairs among Macro6 (SPP1^hi^ macrophages) and Fibro3 (RCFs) (human). **E** GO enrichment analysis of linkage-related genes between SPP1^hi^ macrophages and RCFs. **F** Immunofluorescence staining of CD68 and TNF on mouse cardiac MI tissue. Scale bars: 20 µm. **G** Immunofluorescence staining of CD68 and IL1B on mouse cardiac MI tissue. Scale bars: 20 µm

Given that we saw some variability in the interactions between macrophages and fibroblasts in MI and ICM, we believed that the interactions between different macrophage subpopulations and fibroblast subpopulations also varied. To decipher the receptor-ligand interactions in different macrophage and fibroblast subsets, we introduced the CellPhoneDB ligand-receptor complexes database to calculate the interactions between them. To better distinguish between mouse and human fibroblast and macrophage subpopulations, we named mouse fibroblast cluster0-cluster11 as fibro0-fibro11 and human fibroblast cluster0-cluster11 as Fibro0-Fibro11. The mouse macrophage subpopulations cluster0- cluster7 were named macro0-macro7, and the human macrophage subpopulation cluster0-cluster8 were named Macro0-Macro8. For the mouse scRNA-seq dataset, we observed a total of 301 interactions of macrophages with fibroblasts during the acute phase of MI (Fig. 8A). There were 24 significant interactions between macro0 (Gpnmb^+^Fabp5^+^macrophage) and fibro5 (mouse RCFs), including TNFSF12-TNFSF12A, CD74-APP and CD74-COPA (Fig. 8B). There were a total of 29 significant interactions between macro0 and fibro10 (Mki67^+^ fibroblasts), including CCR5-CCL7, TGFB1-TGFbeta receptor1, TNFSF12-TNFRSF12A and SPP1-CD44 (Fig. 8C). Integration of scRNA-seq datasets from various tissues and organs revealed that SPP1^+^ macrophages increase during fibrotic disease in various human tissues [50]. It is important to note that LGALS9 may be extensively involved in the interaction of macro0 and macro1 with different fibroblast subpopulations, as shown by CellphoneDB results (Fig. 8B and 8C; Figure S7A and S7B). Next, we analyzed the interaction between fibroblast subpopulations and macrophage subpopulations under different disease stages of MI in humans (Fig. 8A; Figure S7C through S7F). Cellphonedb analysis showed a total of 107 interactions in the MI group, 242 interactions in the control group, and 525 interactions in the ICM group (Fig. 8A). There were 91 interactions between Macro 6 (SSP1^hi^ macrophages) and Fibro 3 (human RCFs) in the MI group, most of which were associated with collagen deposition and ECM production (Fig. 8D and 8E). This is consistent with the rapid activation of fibroblast subpopulations during the acute phase to prevent cardiac rupture by repairing damaged areas through collagen deposition. Of note, we found that FN1 is extensively involved in macrophage-fibroblast interaction in MI (Figure S7A). However, these connections were reduced in the control and ICM groups, suggesting that FN1 may be involved in macrophage activation of fibroblasts in early stage after MI injury. There were 44 interactions between Macro 1 and Fibro 6 in the ICM group. Among these interactions, we identified a number of TNF-related interactions, which, combined with the results of previous NicheNet analyses, suggest that IL1B and TNF signaling play an important role in fibroblast-macrophage communication in the MI and ICM. Therefore, we explored whether inflammatory macrophages were present in the infarct zone in the later stages of the disease. Immunofluorescence results showed that some macrophages expressing TNF and IL1B were still present in the infarcted area 28 days after MI (Fig. 8F and 8G), a result suggesting that persistent inflammation is still present in the scarred area.

## Discussion

Fibrosis is characterized by the deposition of collagen and other ECM molecules, in which activated fibroblasts play an important role. Fibrosis is an important cause of organ and tissue dysfunction in many chronic diseases [51,52]. Cardiac fibrosis is an important part of cardiac remodeling, accompanied by changes in the extracellular matrix and interaction with a variety of mesenchymal and immune cells. The heterogeneity of cardiac fibroblasts is described in several articles using scRNA-seq, including MI, DCM, and HCM.

Our study identified fibroblast subpopulations with distinct transcriptional phenotypes by integrating multiple MI scRNA-seq datasets. We identified a total of four fibroblast subpopulations that increased substantially after MI, including cluster 5 (RCFs), cluster 7 (matrifibrocytes), cluster 9 (Ccl2^+^ fibroblasts) and cluster 10 (Mki67^+^ fibroblasts). The high expression of Mki67 in cluster 10 compared to the other fibroblast subsets represents its high proliferative activity. This is corroborated by the results of CytoTRACE that cluster 10 has a greater differentiation potential compared to other fibroblasts. Cluster 5 high expression of Postn and Acta2 represents activated fibroblasts, and the results of GO enrichment analysis also focused on ECM formation and collagen deposition. In addition, cluster 5 highly expressed Cthrc1, which is consistent with the results of a previous study that a fibroblast subpopulation activated during the acute phase of MI represents reparative cardiac fibroblasts. The results of RNA velocity analysis and PAGA graph abstraction suggest that RCFs were mainly derived from Mki67^+^ fibroblasts. Genes highly expressed in cluster 7 included Comp, Wisp2, Ctgf and Meox1, some classical marker genes for myofibroblasts, represent matrifibrocytes that appear in the late stages of MI. GO enrichment showed that these genes were predominantly enriched in the ECM. We hypothesize that, unlike RCFs, this subset of fibroblasts, which appears in the later stages of MI, may be involved in the persistent inflammation and fibrosis in cardiac tissues and have an important role in pathological myocardial remodeling.

Given that the CTHRC1 gene ranks first in RCFs in acute MI, we were interested in exploring CTHRC1 gene expression in a model of chronic fibrosis and heart failure. CTHRC1 expression was also elevated in a TAC-induced heart failure model, as well as in a mouse model three months after MI, and these results suggest that CTHRC1 may be persistently expressed in a subset of fibroblasts. Some fibroblasts in both DCM and HCM also expressed CTHRC1. Interestingly, fibroblasts with high expression of CTHRC1 also expressed high levels of POSTN, further demonstrating that this is an activated class of fibroblasts. CTHRC1-expressing fibroblasts have been found in other diseases including keloids [44], lung injury caused by COVID-19 [53], prostate cancer [54] and idiopathic pulmonary fibrosis (IPF) [55]. In lung disease, CTHRC1^+^ fibroblasts exhibit a pro-fibrotic population and expand in damaged lung tissue and are characterized by high expression of collagen along with TGFB1 [55,56]. According to the results of our scRNA-seq datasets analysis, CTHRC1^+^ fibroblasts also have a pro-fibrotic phenotype in acute and chronic cardiac injury, but this fibroblasts with ECM deposition have different outcomes in different states of the disease, such as MI.

Many studies summarize the relationship between fibrosis and macrophages, and the most recently mentioned SAMs are a subpopulation of macrophages expressing CD9 and TREM2 [7,8]. In liver fibrosis, different types of macrophages have different roles, and they have both fibrosis-promoting and matrix degradation roles [57]. In another liver fibrosis study, Liu et al. found that fibrolytic SAM-derived CXCL9 in turn leads to extracellular matrix degradation through MMP13 production [58]. In human liver cirrhosis, TREM2^+^CD9^+^ macrophages, exhibit a pro-fibrotic phenotype and are spatially located close to the scarred areas of collagen deposition [8]. A subpopulation with high expression of CD9 and TREM2 was also identified in our study, we also found that a subset of TREM2^+^CD9^+^ macrophages highly expressed Gpnmb, Fabp5, Ctsd, Cd63 and Spp. Cardiac Gpnmb^+^Fabp5^+^ macrophages began to increase on day 3 after MI, peaked on day 7, and decreased dramatically on day 14, a trend consistent with RCFs. In a larger CD45^+^ scRNA-seq dataset we observed similar findings, with Gpnmb^+^Fabp5^+^ macrophages appearing in the acute phase but decreasing 14 days after MI. However, different results were obtained in our integrated human dataset, where the population characterizing Gpnmb^+^Fabp5^+^ macrophages appeared in cardiac tissue from ICM patients but not in MI cardiac tissue. Nevertheless, concordant with the results obtained in the mouse MI dataset, macrophages highly expressing SPP1 were predominantly found in MI tissues. Trajectory analysis indicates that this macrophage is derived from monocytes rather than resident macrophages, consistent with previous findings [47,59].

We then attempted to study macrophage-fibroblast communication using prevailing analytical tools including NicheNet and CellphoneDB. Consistent with previous findings [13], we found that TGFB1 has an important role in macrophage-fibroblast interactions in both the MI and ICM groups. In addition to this, we discover that macrophage-derived TNF and IL1B may be involved in fibroblast-macrophage cross-talk in ICM and MI disease states. Immunofluorescence staining results supported the persistence of a few inflammatory macrophages in the scarred area. Interaction analysis between cellular subsets indicates differences in linkages between different subpopulations. Among them, LGALS9 connections were present in a variety of fibroblast-macrophage interactions. LGALS9 has been shown to have a significant relationship with hepatoblastoma [60], liver cancer [61] and chronic myeloid leukemia [62] in some previous single-cell studies. Here we hypothesize that LGALS9 has an association with MI and may serve as a diagnostic and prognostic indicator, but subsequent studies are needed to prove that. We observed that the number of connections between RCFs and SSP1^hi^ macrophages far exceeded the number of other fibroblast-macrophage connections, and GO enrichment analysis of these genes focused on collagen deposition and ECM generation. These results suggest that a subset of macrophages are involved in scar formation and maturation during MI, further extending the close relationship between SPP1 macrophages and fibrosis.

Considering the time specificity of the different fibroblast and macrophage subpopulations, a few cell subpopulations may have diagnostic and therapeutic value. For example, matrifibrocytes are predominantly found in the late stages of MI, and previous studies have shown that these cells are predominantly found in the scar area and express Comp, Cilp, and Meox1. A previous study suggested that Cilp could serve as a novel biomarker for cardiac fibrosis [63]. We believe that constructing gene-regulatory networks (GRNs) based on DEGs and TFs from this subset may provide a more effective diagnostic and therapeutic approach for cardiac fibrosis. We also observed that the number of Lyve1^+^ macrophage declined rapidly after MI. This macrophage has also been defined as a cardiac resident macrophage with a protective role in cardiac physiological and pathological states. Therefore, increasing the number of this macrophage type during MI may improve cardiac repair and thus have potential therapeutic value. For example, a recent study suggests that resident macrophage (MerTK + macrophage) transfer rescues impaired cardiac repair^64^.

## Limitations of the study

Our study comprehensively depicts the dynamics of fibroblasts and macrophages by integrating single-cell datasets from multiple studies. But there are several limitations in this study. First, we integrated multiple datasets and utilized additional datasets to validate our findings. Despite employing various methods, such as the harmony package, to mitigate discrepancies, we were still impacted by variability between datasets. Second, we experimented in vitro to validate the role of CTHRC1. However, additional in vivo experiments are needed to further investigate the specific role of CTHRC1 in diseases such as heart failure. Overall, we expect that our findings at the single-cell resolution will provide further insights into the relationship between macrophages and fibrosis, and lead to new directions in the diagnosis and treatment of cardiac fibrosis.

## Conclusions

In conclusion, our work provides a comprehensive overview of the dynamics of fibroblast and macrophage subsets after MI. Analyze and characterize the signatures and differentiation trajectories of several macrophages and fibroblasts subsets that emerge after MI and explore the conserved of these signatures in human. We found that CTHRC1^+^ fibroblasts represent a repair cell in the acute phase of MI, whereas in the chronic disease state it represents an activated fibroblast involved in pathological fibrosis of the heart. Finally, we analyzed the interaction between fibroblasts and macrophages in the state of MI and ICM. We showed that macrophage-derived TGFB1, TNF and IL1B have important roles in interaction with fibroblasts and identified interactions between RCFs and SPP1^hi^ macrophages involved in collagen deposition and extracellular matrix generation. Our study provides a valuable reference for understanding the relationship between fibrosis and immunity in MI.

### Supplementary Information

Supplementary material 1.

## Data Availability

The datasets used and analyzed in this study are available from the Gene Expression Omnibus database (https://www.ncbi.nlm.nih.gov/geo/), ArrayExpress (https://www.ebi.ac.uk/biostudies/arrayexpress), Figshare (https://figshare.com/) and Human Cell Atlas (https://www.humancellatlas.org/).
