# Supplementary material for: Macrophage and fibroblast trajectory inference and crosstalk analysis during myocardial infarction using integrated single-cell transcriptomic datasets
Source: J Transl Med. 2024 Jun 12;22:560. doi: 10.1186/s12967-024-05353-x (PMC11167890; doi:10.1186/s12967-024-05353-x)
Supplement: Supplementary file 1 — Supplementary material 1. [file 12967_2024_5353_MOESM1_ESM.pdf]

SUPPLEMENTAL MATERIAL

This file includes:

Supplemental Figure 1-7

Supplemental Table 1-6

Supplemental Figures

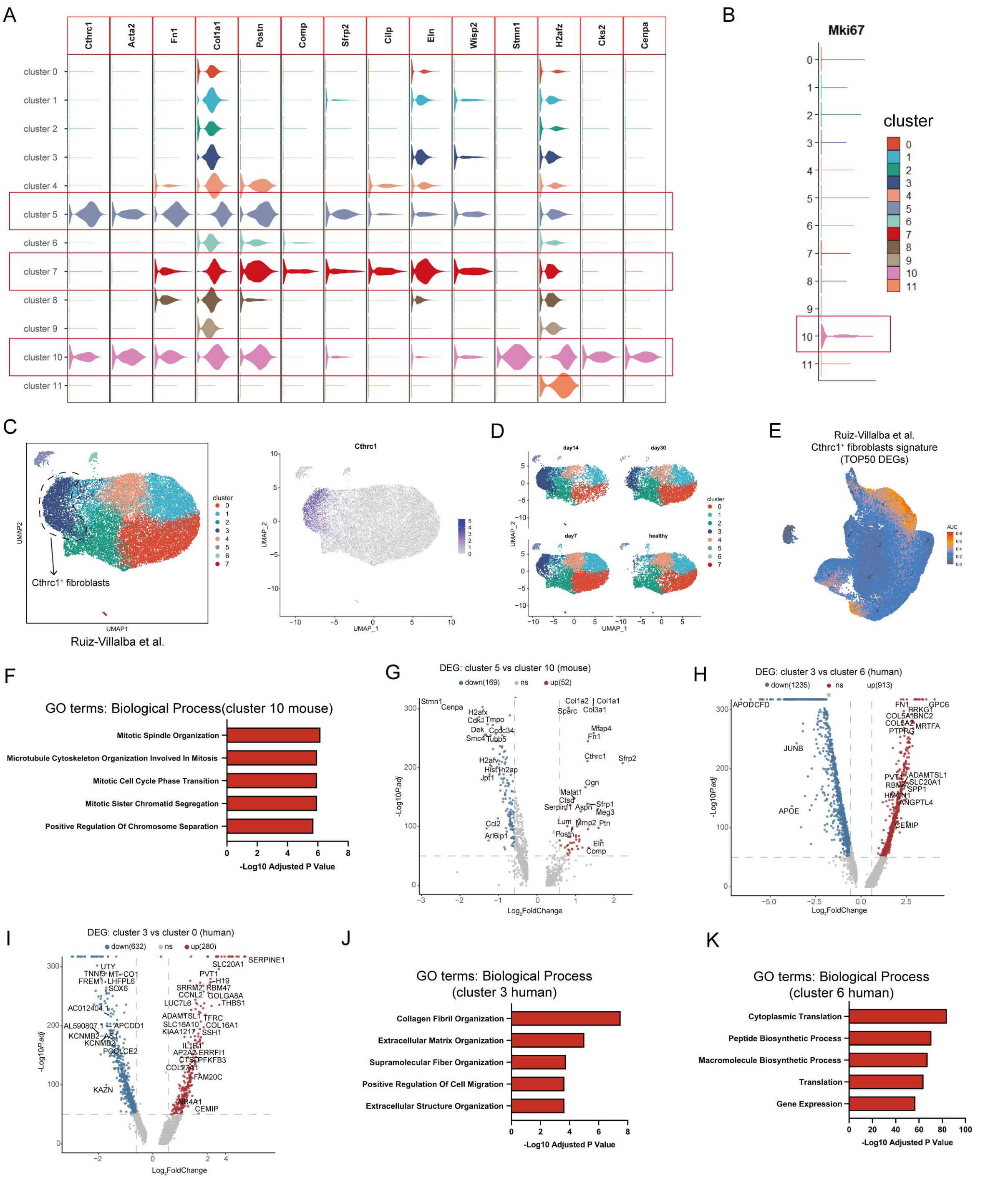

Figure S1. Fibroblast subpopulations have differential expression profiles. A Violin plots showing the expression of the top 5 genes in cluster 5, cluster 7 and cluster 10. **B** Violin plots showing the expression of Mki67 in fibroblasts clusters. **C**

UMAP plot of fibroblasts from the Ruiz-Villalba et al. generated scRNA-seq dataset. A total of 8 clusters were identified. **D** UMAP plots of fibroblasts from different groups colored by cluster. **E** UMAP plots showing mouse Cthrc1^{+} fibroblasts signatures using the top 50 DEGs from Cthrc1^{+} fibroblasts and mapped into fibroblasts from our generated datasets. **F** GO enrichment analysis of cluster 10 (mouse) top 50 genes. **G** The volcano plots showing the DEGs between cluster 5 vs. cluster 10 (mouse). **H** The volcano plots showing the DEGs between cluster 3 vs. cluster 6 (human). **I** The volcano plots showing the DEGs between cluster 3 vs. cluster 0 (human). **J** GO enrichment analysis of cluster 3 (human) top 50 genes. **K** GO enrichment analysis of cluster 6 (human) top 50 genes.

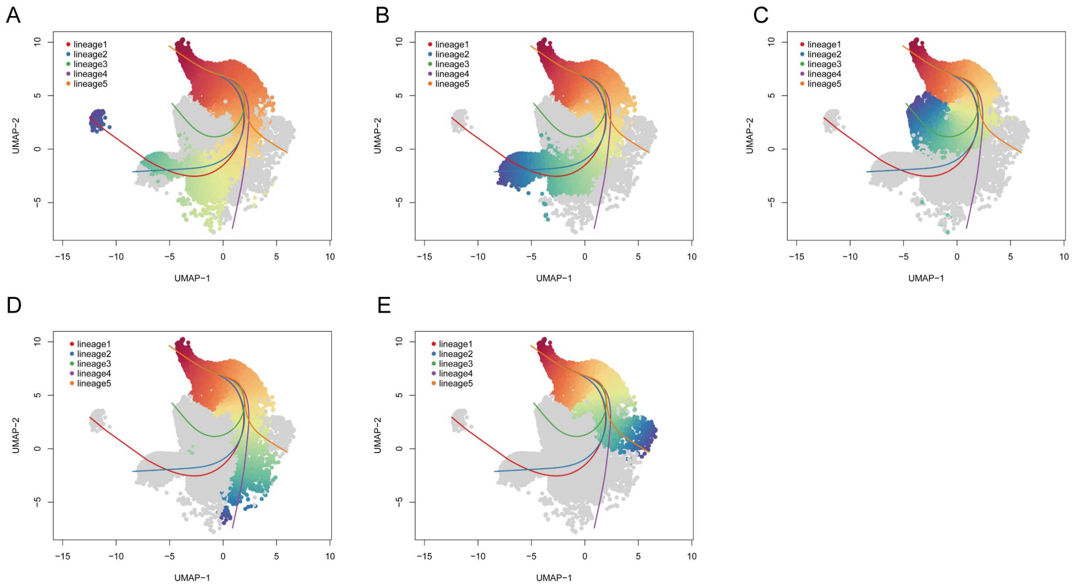

Figure S2. The differentiation trajectory of fibroblast subsets in the acute phase after MI. (**A-E**) Slingshot differentiation trajectory analyses of fibroblast subsets in the lineage 1 (**A**), lineage 2 (**B**), lineage 3 (**C)**, lineage 4 (**D**), and lineage 5 (**E**). The predicted trajectories are drawn on UMAP projection of fibroblasts.

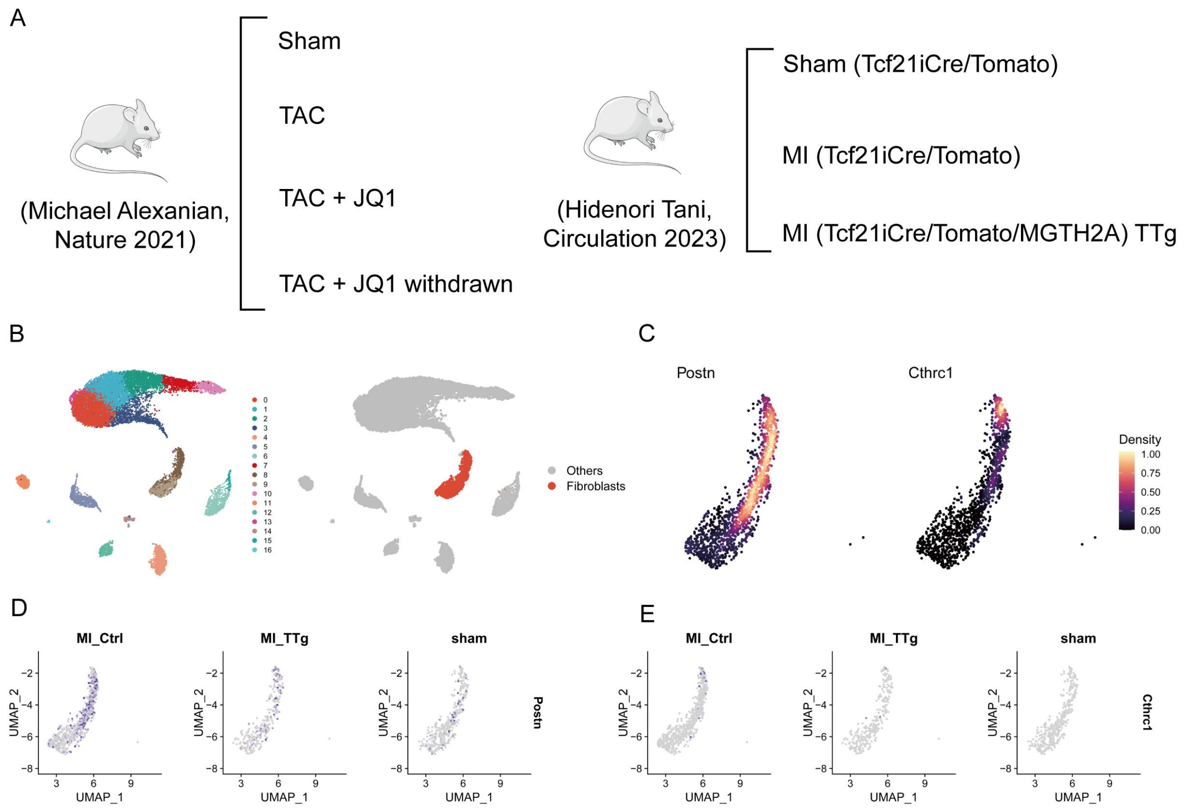

Figure S3. Characterization of CTHRC1⁺ fibroblasts in chronic MI. A Different experimental groupings for each of the two mouse scRNA-seq datasets. The dataset generated by Michael Alexanian is divided into four groups, while the dataset generated by Hidenori Tani is divided into three groups. **B** UMAP plot showing the fibroblast. All other types of cells were colored in gray. **C** Gene expression of Postn and Cthrc1. **D** Expression by samples of Postn shown as feature plots in fibroblasts. **E** Expression by samples of Cthrc1 shown as feature plots in fibroblasts.

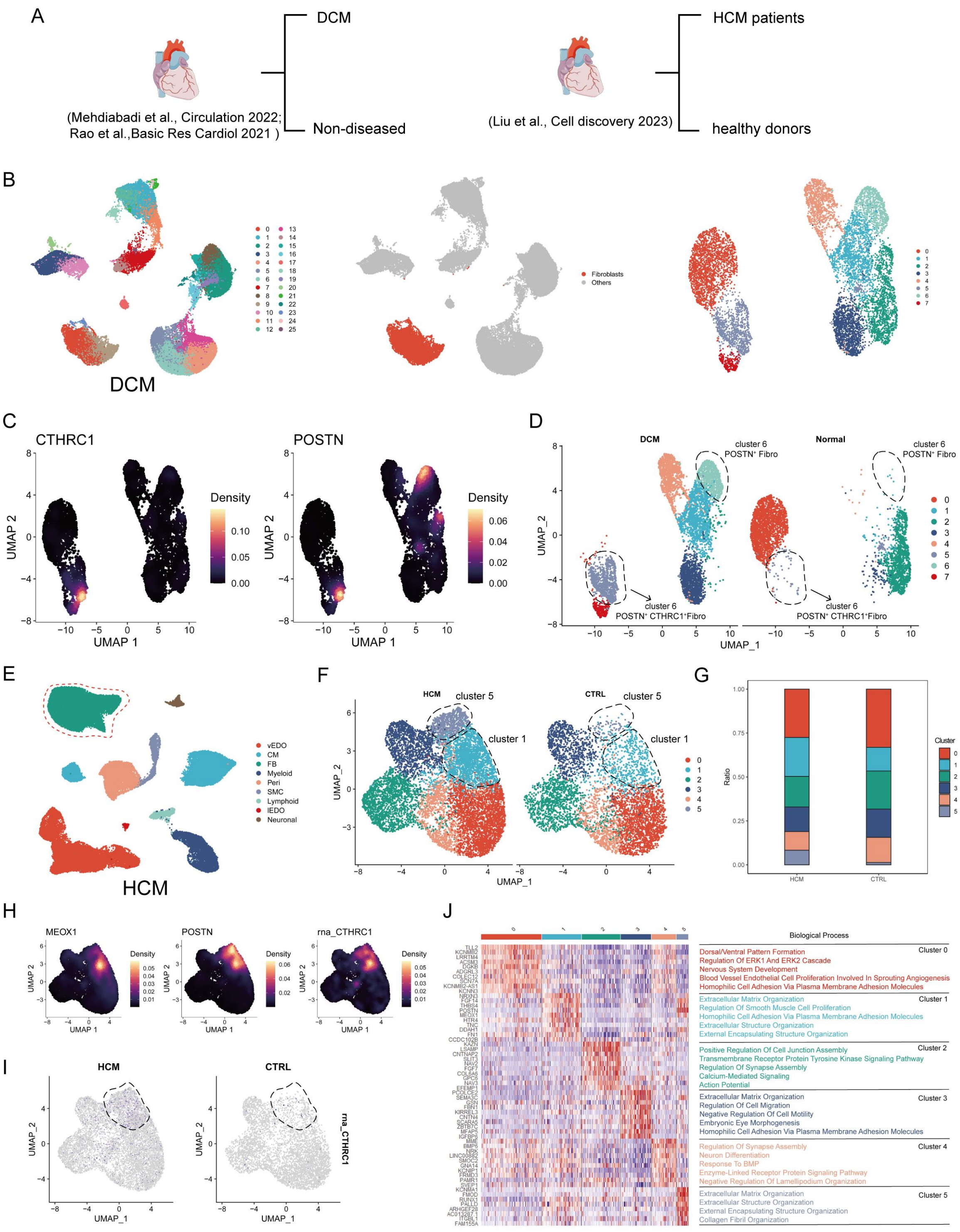

Figure S4. CTHRC1⁺ fibroblasts represent a subset of fibroblasts highly expressing the POSTN gene in DCM and HCM. A Different experimental groupings for the DCM and HCM scRNA-seq datasets. **B** UMAP plot showing the

fibroblast and their subclusters. A total of 8 clusters were identified. All other types of cells were colored in gray. **C** Gene expression of Postn and Cthrc1. **D** UMAP plots of fibroblast from different samples colored by cluster. **E** UMAP plot of all cells from the human HCM scRNA-seq dataset colored by cell type. **F** UMAP scRNA-seq plot of fibroblasts from the human HCM scRNA-seq dataset. A total of 6 clusters were identified. **G** Fraction of different cell types from control and HCM group. **H** Gene expression of MEOX1, POSTN and CTHRC1. **I** Expression by sample of CTHRC1 shown as feature plots in fibroblasts. **J** Heatmap showing the expression profiles of top 10 genes ranked by LogFC of each cluster (left). Enriched GO terms for marker genes of each cluster (right).

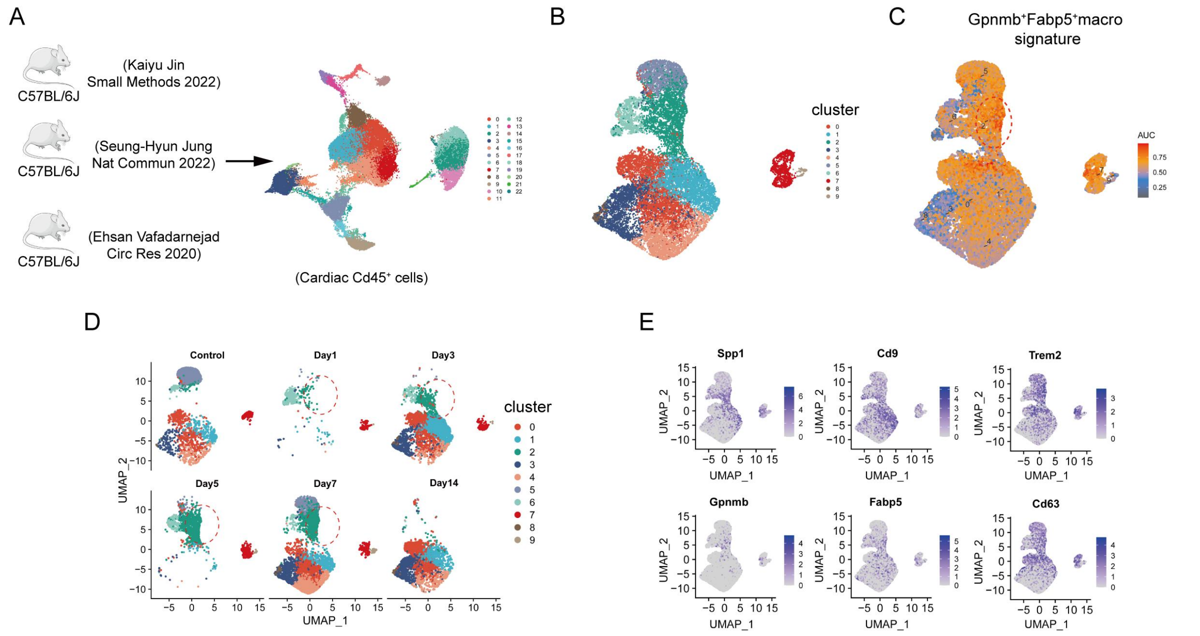

Figure S5. Validation of Gpnmb⁺Fabp5⁺ macrophage characterization in another integrated mouse CD45⁺ scRNA-seq dataset. A A large CD45⁺ scRNA-seq dataset that was obtained by integrating the dataset generated by Kaiyu Jin, the dataset generated by Seung-Hyun Jung and the dataset generated by Ehsan Vafadarnejad (left). UMAP plots of cells from different samples colored by cluster (right). **B** UMAP scRNA-seq plot of macrophages from the large CD45⁺ scRNA-seq dataset. A total of 10 clusters were identified. **C** UMAP plots showing mouse Gpnmb⁺Fabp5⁺ macrophages signatures using the top 30 DEGs from mouse Gpnmb⁺Fabp5⁺ macrophages and mapped into macrophages from the large CD45⁺ scRNA-seq dataset. **D** UMAP plots of macrophages from control, 1, 3, 7 and 14 days after MI colored by clusters. Cluster 2 is marked by a red circle. **E** Expression of Spp1, Cd9, Trem2, Gpnmb, Fabp5 and Cd63 shown as feature plots in macrophages.

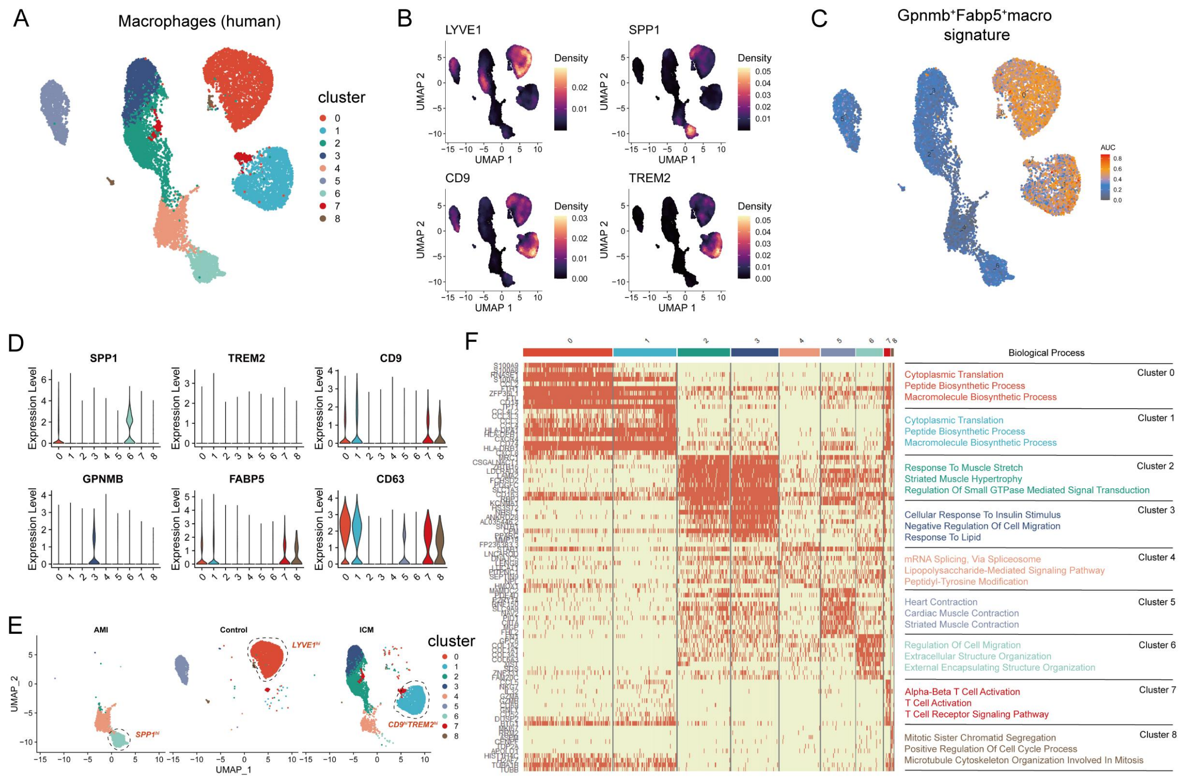

Figure S6. Gpnmb⁺Fabp5⁺ macrophage signature are conserved in human heart. A UMAP scRNA-seq plot of macrophages from the integrated human scRNA-seq dataset. A total of 9 clusters were identified. **B** Gene expression of LYVE1, SPP1, CD9 and TREM2. **C** UMAP plots showing mouse Gpnmb⁺Fabp5⁺ macrophages signatures using the top 30 DEGs from mouse Gpnmb⁺Fabp5⁺ macrophages and mapped into macrophages from the human scRNA-seq dataset. **D** Violin plot for SPP1, TREM2, CD9, GPNMB, FABP5 and CD63 expression in all cell clusters. **E** UMAP plots of macrophage from different groups colored by cluster. **F** Heatmap showing the expression profiles of top 10 genes ranked by LogFC of each cluster (left). Enriched GO terms for marker genes of each cluster (right).

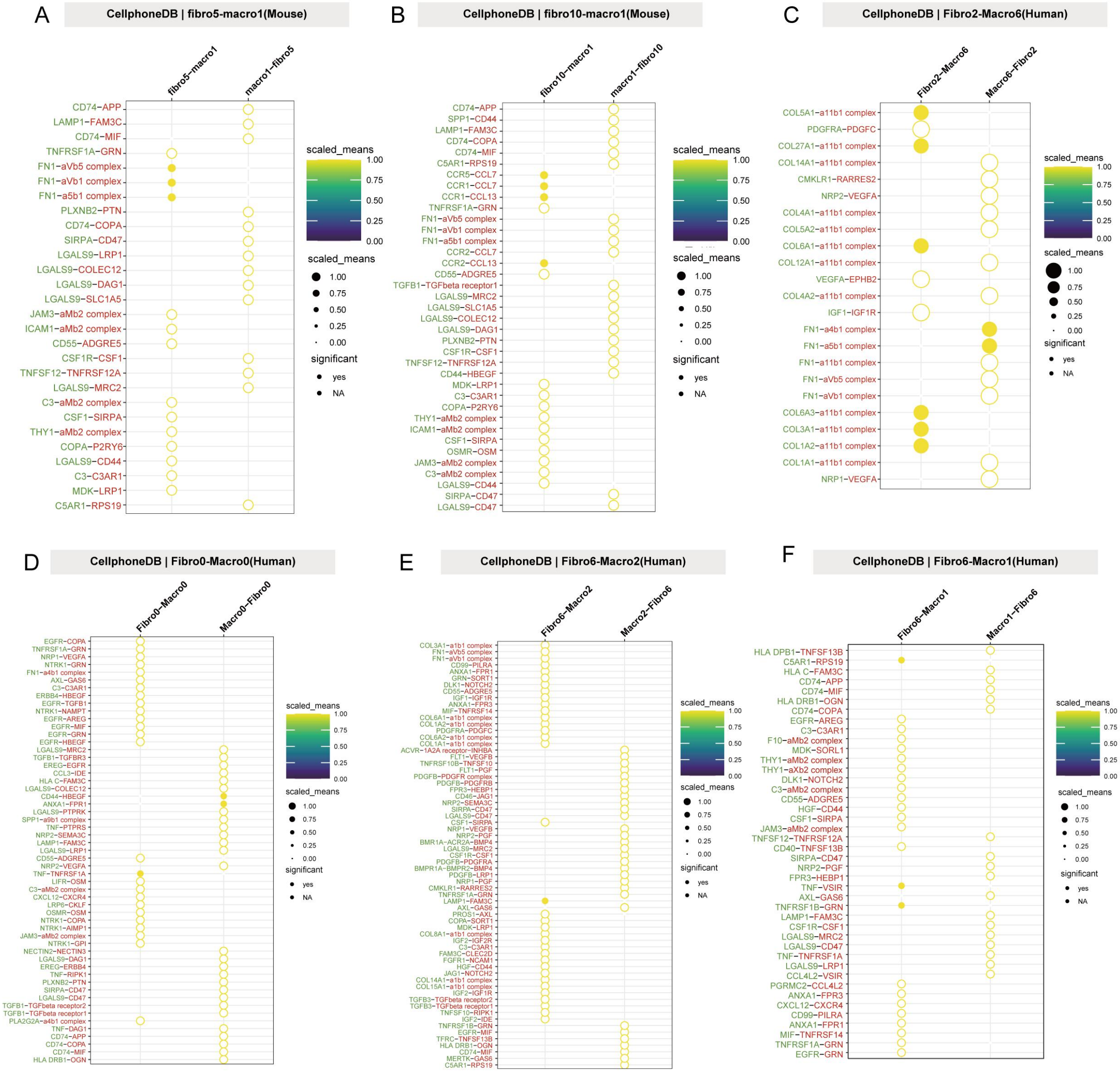

Figure S7. Interaction between macrophage subsets and fibroblast subsets. A Dot plot showing the ligand-receptor pairs among fibro5 and macro1 (Mouse). **B** Dot plot showing the ligand-receptor pairs among fibro10 and macro1 (Mouse). **C** Dot plot showing the ligand-receptor pairs among Fibro2 and Macro6 (Human). **D** Dot plot showing the ligand-receptor pairs among Fibro0 and Macro0 (Human). **E** Dot plot showing the ligand-receptor pairs among Fibro6 and Macro2 (Human). **F** Dot plot showing the ligand-receptor pairs among Fibro6 and Macro1 (Human).

Supplemental Tables

Table S1: The information of the main datasets.

Datasets	Sample name	Injury/Disease status	Strain/Background/Genotype	Cell type
E-MTAB-9816	Sample 1	normal	Cdh5-CreERT2/+; mT/mG	non-cardiomyocyte cells
	Sample 2	1 days post MI	Cdh5-CreERT2/+; mT/mG	non-cardiomyocyte cells
	Sample 3	3 days post MI	Cdh5-CreERT2/+; mT/mG	non-cardiomyocyte cells
	Sample 4	7 days post MI	Cdh5-CreERT2/+; mT/mG	non-cardiomyocyte cells
	Sample 5	14 days post MI	Cdh5-CreERT2/+; mT/mG	non-cardiomyocyte cells
	Sample 6	28 days post MI	Cdh5-CreERT2/+; mT/mG	non-cardiomyocyte cells
E-MTAB-7895	MF17004	sham	C57BL/6J	Cardiac interstitial cells
	MF17008	sham	C57BL/6J	Cardiac interstitial cells
	MF17010	none	C57BL/6J	Cardiac interstitial cells
	MF17013	1 days post MI	C57BL/6J	Cardiac interstitial cells
	MF17014	3 days post MI	C57BL/6J	Cardiac interstitial cells
	MF17015	5 days post MI	C57BL/6J	Cardiac interstitial cells
	MF17016	7 days post MI	C57BL/6J	Cardiac interstitial cells
	MF17017	14 days post MI	C57BL/6J	Cardiac interstitial cells
	MF17018	28 days post MI	C57BL/6J	Cardiac interstitial cells

GSE145154	GSM4307515	Normal	Human	CD45+ cells
	GSM4307516	Normal		CD45- cells
	GSM4307530	ICM		CD45+ cells
	GSM4307531	ICM		CD45- cells
	GSM4307535	ICM		CD45+ cells
	GSM4307536	ICM		CD45- cells
	GSM4307540	ICM		CD45+ cells
	GSM4307541	ICM		CD45- cells
Christoph Kuppe et al. dataset	CK161	AMI	Human	cardiac cells
	CK369	AMI		cardiac cells
	CK373	AMI		cardiac cells
	CK375	AMI		cardiac cells
	CK376	AMI		cardiac cells
	CK158	Control		cardiac cells
	CK367	ICM		cardiac cells
	CK368	ICM		cardiac cells
	CK370	ICM		cardiac cells

Table S2: The information of the validation datasets.

Datasets	Accession number	Injury/Disease status	Strain/Background/Genotype	Cell type
GSE132144	GSM3847600	Healthy Myocardium	C57BL/6	Col1a1-GFP+ cells
	GSM3847601	7 days post MI	C57BL/6	Col1a1-GFP+ cells
	GSM3847602	14 days post MI	C57BL/6	Col1a1-GFP+ cells
	GSM3847603	30 days post MI	C57BL/6	Col1a1-GFP+ cells
GSE185265	GSM5609416	Sham	Tcf21iCre/Tomato	non-cardiomyocyte cells
	GSM6607334	Sham2	Tcf21iCre/Tomato	non-cardiomyocyte cells
	GSM5609417	Ctrl-MI (3 months post MI)	Tcf21iCre/Tomato	non-cardiomyocyte cells
	GSM5609418	TTg-MI (3	Tcf21iCre/Tomat	non-cardiomyocyte

		months post MI)	o/MGTH2A	cells
GSE155882	GSM4715045	Sham rep1	C57BL/6	non-cardiomyocyte cells
	GSM4715046	Sham rep2	C57BL/6	non-cardiomyocyte cells
	GSM4715047	TAC rep1	C57BL/6	non-cardiomyocyte cells
	GSM4715048	TAC rep2	C57BL/6	non-cardiomyocyte cells
	GSM4715049	TAC JQ1 rep1	C57BL/6	non-cardiomyocyte cells
	GSM4715050	TAC JQ1 rep2	C57BL/6	non-cardiomyocyte cells
	GSM4715051	TAC JQ1 withdrawn rep1	C57BL/6	non-cardiomyocyte cells
	GSM4715052	TAC JQ1 withdrawn rep2	C57BL/6	non-cardiomyocyte cells
GSE135310	GSM4005123	Steady state	C57BL/6	CD45+ total leukocytes
	GSM4005125	1 days post MI	C57BL/6	CD45+ total leukocytes
	GSM4005126	3 days post MI	C57BL/6	CD45+ total leukocytes
	GSM4005127	5 days post MI	C57BL/6	CD45+ total leukocytes
	GSM4005124	7 days post MI	C57BL/6	CD45+ total leukocytes
GSE163129	GSM4972357	Steady-state	C57BL/6J	CD45+ leukocytes
	GSM4972358	1 days post MI	C57BL/6J	CD45+ leukocytes
	GSM4972359	3 days post MI	C57BL/6J	CD45+ leukocytes
	GSM4972360	5 days post MI	C57BL/6J	CD45+ leukocytes
	GSM4972361	7 days post MI	C57BL/6J	CD45+ leukocytes
GSE163465	GSM4985022	Sham	C57/BL	Cd45+ immune cell
	GSM4985023	3 days post MI	C57/BL	Cd45+ immune cell
	GSM4985024	7 days post MI	C57/BL	Cd45+ immune cell
	GSM4985025	14 days post MI	C57/BL	Cd45+ immune cell
	GSM5513773	3 days post MI	C57/BL	Cd45+ immune cell
	GSM5513774	7 days post MI	C57/BL	Cd45+ immune cell
	GSM5513775	14 days post MI	C57/BL	Cd45+ immune cell
GSE185100	GSM5606407	DCM	Human	cardiac cells

	GSM5606408	DCM		cardiac cells
	GSM5606409	DCM		cardiac cells
	GSM5606413	nondiseased		cardiac cells
	GSM5606414	nondiseased		cardiac cells
	GSM5606415	nondiseased		cardiac cells
GSE145154	GSM4307515	Normal	Human	CD45+ cells
	GSM4307516	Normal		CD45- cells
	GSM4307520	DCM		CD45+ cells
	GSM4307521	DCM		CD45- cells
	GSM4307525	DCM		CD45+ cells
	GSM4307526	DCM		CD45- cells
Liu et al. dataset	(https://doi.org/10.6084/m9.figshare.c.5777948.v2).	HCM and healthy	Human	cardiac cells

Table S3: The information of the primary antibodies used in immunofluorescence staining.

Antibody description	Company	No. and dilution
CTHRC1	Proteintech	#16534-1-AP (1:100)
POSTN	Proteintech	#19899-1-AP (1:100)
Collagen I	Proteintech	#14695-1-AP (1:100)
Collagen III	Proteintech	#22734-1-AP (1:100)
CD68	Abcam	#ab955 (1:100)
TNF	Cell Signaling Technology	#11948 (1:100)
IL1B	Abmart	#TA5103M (1:100)

Table S4: The information of the primers used in RT-PCR.

Target	Forward Sequences (5'→3')	Reverse Sequences (5'→3')
Rat-Gapdh	GACATGCCGCCTGGAGAAAC	AGCCCAGGATGCCCTTTAGT

Rat-Col1a1	GAGAGAGCATGACCGATGGATT	TGGACATTAGGCGCAGGAA
Rat-Col3a1	GTGGTCCTCCAGGAGAAAATGGAAA	GCACCCGCACCGCCTGGCTCAC
Rat-Ctgf	GGAAGACACATTTGGCCCTG	GCAATTTTAGGCGTCCGGAT
Rat-Cthrc1	TGTCCGTCGTGGATCTGAC	CCTGCTTCACCACCTTCTTG
Rat-Fn1	GGATCCCCTCCCAGAGAAGT	GGGTGTGGAAGGGTAACCAG
Rat-Postn	TGCAAAAAGACACACCTGCAA	GGCCTTCTCTTGATCGCCTT

Table S5: The top 50 DEGs of RCFs, Matrifibrocytes, Ccl2$^+$ fibroblasts and Mki67$^+$ fibroblasts.

Cluster 5 (RCFs)	Cluster 7 (Matrifibrocytes)	Cluster 9 (Ccl2$^+$ fibroblasts)	Cluster 10 (Mki67$^+$ fibroblasts)
Cthrc1	Comp	Mt2	Stmn1
Acta2	Sfrp2	Ccl2	H2afz
Fn1	Cilp	Timp1	Cks2
Col1a1	Eln	Mt1	Cenpa
Postn	Wisp2	Prg4	Acta2
Tagln	Ctgf	Angptl4	Hmgb2
Ddah1	Angptl7	Ccl7	Pclaf
Ptn	Itgbl1	Serpina3n	Birc5
Col1a2	Pmepa1	Ptx3	Selenoh
Sfrp2	Cst3	Mif	Cdc20
Csrp2	Meox1	Ldha	Tubb5
Lox	Mfap4	Slc25a5	Timp1
Col3a1	1500015O10Rik	Pdpn	Ube2s
Tpm2	Col8a1	Tnfrsf12a	Ube2c
Myl9	Itgb5	Nme1	Ran
Sparc	Emp1	Eif5a	Cdca8
Col5a2	Adamtsl2	Eif4a1	Hmgn2
Timp1	Ltbp2	Itga5	Ccnb2
Palld	Fibin	Hspa5	Tuba1b
Plac8	Aspn	Pgk1	Cdca3
Wisp1	Frzb	Ncl	Tpm2

Marcksl1	Postn	Ran	Spp1
Ltbp2	Fxyd6	Srm	Top2a
Mfap4	Pam	Hspd1	Cdk1
C1qtnf6	Cd200	Rbm3	Cks1b
Actb	Cfh	Manf	Spc24
Slc39a1	Crlf1	Plin2	Tk1
Ppic	Tgfb3	Nhp2	Tagln
Rflnb	Cst6	Sod2	H2afx
Lgals1	Adamts2	Smg6	Tubb6
Kdelr2	Lbh	Aldh1a2	Tagln2
Serpinh1	Mmp2	Gm12840	Tubb4b
Actn1	Pdgfrl	Ppa1	Prc1
Sfrp1	Igfbp7	Lrrc59	Cenpf
Crlf1	Ltbp4	Eif4ebp1	Pbk
Aspn	Col3a1	Rps2	Lockd
Lbh	Col4a1	C1qbp	Lgals1
Loxl3	Ckb	Ppp1r14b	Cenpm
Mdk	F2r	Gar1	Actb
Isg15	Adcy7	Ebna1bp2	Smc2
Vamp5	Nbl1	Fkbp11	Cthrc1
Wisp2	Col5a2	Tomm40	Smc4
Mfap2	Rflnb	Ddx39	Tyms
Col14a1	Fstl1	Mrto4	Tpx2
Col8a1	Col4a2	Timm8a1	Rrm2
Fstl1	Col4a3	Eef1e1	Ccna2
Col5a1	Mfap5	Tuba1c	Ccnb1
Ctsd	Tmem119	Rps18	Cdkn3
1500015O10Rik	Gxylt2	Rplp0	Ckap4
Mmp14	Bgn	Eif6	Tpm4

Table S6: The top 50 DEGs of Gpnmb$^+$Fabp5$^+$ macrophage, Arg1$^+$macrophage, Il1b$^+$ macrophage and Lyve1$^+$ macrophage.

Cluster 0 (Gpnmb+Fabp5+ macrophage)	Cluster 1 (Arg1+macrophage)	Cluster 2 (Il1b+ macrophage)	Cluster 3 (Lyve1+ macrophage)
Gpnmb	Arg1	H2-Ab1	Hspa1a
Fabp5	Ccl6	H2-Aa	Mgl2
Ctsd	Chil3	Cd74	Folr2
Syngr1	Ccl9	H2-Eb1	Klf2
Trem2	Fn1	Tmem176b	Jun
Ms4a7	Ccl2	Cd83	Cbr2
Ctsb	Ccl7	Cd72	H2-Eb1
Cd63	Srgn	Il1b	Serinc3
Apoe	Mif	Rpl18a	F13a1
Pld3	Pgk1	Cst3	Cd81
Lgals3	Thbs1	Rps9	Hpgd
Igf1	Pkm	Tmem176a	H2-Aa
Creg1	S100a4	Cd52	Cd74
Psap	Ldha	H2-DMa	H2-Ab1
Hexa	Eno1	Fau	Fcgrt
Ftl1	Tgfbi	H2-DMb1	Egr1
Cd68	Ltb4r1	Rps27	Tmem176b
Ctss	Cebpb	Rps14	Tmem176a
Timp2	S100a10	C1qb	Hspa1b
Spp1	Slc16a3	Dusp2	Lilra5
Fth1	S100a6	C1qa	Mrc1
Grn	Tpi1	Ccrl2	Txnip
Lyz2	Anxa2	Apoe	Trf
Npc2	Ccr1	Cxcl16	Cfh
Itm2b	Gapdh	Hexb	Ltc4s
C1qb	Cstb	Ighm	St3gal6
Tyrobp	Gda	Rpl37a	Ighm
Selenop	Aldoa	Nfkbia	Selenop
Lamp1	Pgam1	Rps5	C1qc
Wfdc17	Msr1	Cd14	C1qa
Serpinb6a	Plin2	Btg2	Cst3

Fcrls	Nme1	Rpl32	Tmcc1
Cyba	S100a11	Ms4a7	Marcksl1
Lgmn	Eif4a1	Ctsh	Pf4
C1qc	Dok2	Rpl37	Malat1
Ctsz	C5ar1	H2-K1	Mef2c
Ctsa	Pfn1	Rps11	Csf1r
Akr1a1	Calm1	Ly86	Igfbp4
Cxcl16	Capg	Ctss	C1qb
Mpeg1	Ccr2	Rpl26	Jund
C1qa	Plac8	Rpl36	Pid1
Prdx1	Aprt	Rpl17	Klf6
Gyg	Rap1b	Rps29	Nfkbiz
Ccl8	Hilpda	Kdm6b	Kctd12
Ctsh	Cfp	B2m	Dnajb1
Ftl1-ps1	Pdia6	Slamf9	H2-DMa
Cd300c2	Gsr	Rps27a	Maf
Ctsl	Arpc2	Laptm5	Tsc22d3
Tspan4	Prdx6	Unc93b1	Gas6
Ypel3	Emb	Junb	Cd83
